# Branched-chain amino acids alleviate NAFLD via inhibiting *de novo* lipogenesis and activating fatty acid β-oxidation in laying hens

**DOI:** 10.1016/j.redox.2024.103385

**Published:** 2024-10-03

**Authors:** Huafeng Jian, Ru Li, Xuan Huang, Jiankui Li, Yan Li, Jiangang Ma, Mingkun Zhu, Xinyang Dong, Hua Yang, Xiaoting Zou

**Affiliations:** a Institute of Feed Science, College of Animal Sciences, Zhejiang University, Key Laboratory of Molecular Animal Nutrition (Zhejiang University), Ministry of Education, Zhejiang Key Laboratory of Nutrition and Breeding for High-quality Animal Products, Hangzhou, 310058, China; b Jiangsu Key Laboratory of Sericultural Biology and Biotechnology, School of Biotechnology, Jiangsu University of Science and Technology, Zhenjiang, 212100, China; c Key Laboratory of Silkworm and Mulberry Genetic Improvement, Ministry of Agriculture and Rural Affairs, The Sericultural Research Institute, Chinese Academy of Agricultural Sciences, Zhenjiang, 212100, China; d Xianghu Laboratory, Hangzhou, 311231, China; e Institute of Agro‐product Safety and Nutrition, Zhejiang Academy of Agricultural Sciences, Hangzhou, 310021, China

**Keywords:** Branched-chain amino acids, Nonalcoholic fatty liver disease, Tryptophan-ILA-AHR, MAPK9, Ubiquitination

## Abstract

The adverse metabolic impacts of branched-chain amino acids (BCAA) have been elucidated are mediated by isoleucine and valine. Dietary restriction of isoleucine promotes metabolic health and increases lifespan. However, a high protein diet enriched in BCAA is presently the most useful therapeutic strategy for nonalcoholic fatty liver disease (NAFLD), yet, its underlying mechanism remains largely unknown. Fatty liver hemorrhagic syndrome (FLHS), a specialized laying hen NAFLD model, can spontaneously develop fatty liver and hepatic steatosis under a high-energy and high-protein dietary background that the pathogenesis of FLHS is similar to human NAFLD. The mechanism underlying dietary BCAA control of NAFLD development in laying hens remains unclear. Herein, we demonstrate that dietary supplementation with 67 % High BCAA has unique mitigative impacts on NAFLD in laying hens. A High BCAA diet alleviates NAFLD, by inhibiting the tryptophan-ILA-AHR axis and MAPK9-mediated *de novo* lipogenesis (DNL), promoting ketogenesis and energy metabolism, and activating PPAR-RXR and pexophagy to promote fatty acid β-oxidation. Furthermore, we uncover that High BCAA strongly activates ubiquitin-proteasome autophagy via downregulating UFMylation to trigger MAPK9-mediated DNL, fatty acid elongation and lipid droplet formation-related proteins ubiquitination degradation, activating PPAR-RXR and pexophagy mediated fatty acid β-oxidation and lipolysis. Together, our data highlight moderating intake of high BCAA by inhibiting the AHR/MAPK9 are promising new strategies in NAFLD and FLHS treatment.

## 1 Introduction

Nonalcoholic fatty liver disease (NAFLD), also referred to as metabolic -associated fatty liver disease (MAFLD), has become a global epidemic with no approved therapeutic drugs by FDA [1]. It is characterized by the accumulation of lipid droplets (LD) in the liver and accompanied by elevated nonesterified fatty acids (NEFA) and triglycerides (TG), resulting in steatosis and insulin resistance (IR) [2]. Excessive lipid accumulation in intracellular triggers a series of events within hepatocytes, including oxidative stress, autophagy inhibition, endoplasmic reticulum (ER) stress, and chronic inflammation, ultimately leading to hepatocyte death and fibrosis [3]. NAFLD stands out as one of the most prevalent metabolic chronic liver conditions globally, affecting up to 25 % of the world's population and exhibiting a rising incidence [4]. Approximately a quarter of patients with NAFLD develop from simple steatosis to nonalcoholic steatohepatitis (NASH), which over time advance to cirrhosis and hepatocellular carcinoma (HCC), statistically [2,5]. Obesity-related NAFLD serves as a significant risk factor for its prevalence, and patients with NAFLD face heightened risks of developing various systemic and metabolic complications [6]. For example, IR, type-2 diabetes (T2D), cardiovascular diseases, and extrahepatic cancers [6]. The burden of NAFLD is increasing because of its complex pathogenesis, yet there is currently no FDA-approved agent available for NAFLD [5].

Indeed, recent reports have pointed out that amino acid metabolic imbalance has been related to a raised risk and disease severity of obesity and NAFLD [[7], [8], [9], [10]]. For instance, increased circulating levels of branched-chain amino acids (BCAA) including leucine, isoleucine, and valine, in particular, and aromatic amino acids (AAA, i.e., tyrosine, phenylalanine, and tryptophan), have been associated with a raised risk of metabolic diseases, including NAFLD [[11], [12], [13], [14]]. BCAA have garnered significant attention, both because the three BCAA are almost always eaten and combusted together and their impact on the mammalian target of rapamycin (mTOR) pathways, which play a crucial role in linking nutrition to metabolic health and disease [11]. Additionally, circulating levels of BCAA have been positively correlated with obesity, IR, and metabolic disorders in both rodents and humans and are a possible biomarker of NAFLD [[15], [16], [17]]. Long-term intake of high BCAA diets results in hyperphagia, reduced lifespan and obesity-associated NAFLD via promoting *de novo* lipogenesis (DNL) and hepatic steatosis [9]. These adverse impacts are not solely attributed to elevated BCAA levels or the activation of hepatic mTOR but instead contribute to a change in the proportion of dietary BCAA compared to other amino acids, particularly tryptophan and threonine [9]. However, lifelong restriction of BCAA leads to a 30 % increase in lifespan and a decrease in frailty among males, but not include female [18]. The adverse metabolic impacts of BCAA have been confirmed to be induced by isoleucine and valine and isoleucine has the strongest role [19]. Restriction of isoleucine promotes metabolic health by remodeling liver and adipose metabolism, increasing hepatic insulin sensitivity and ketogenesis and promoting energy expenditure, activating the fibroblast growth factor 21 (FGF21)-uncoupling protein 1 (UCP1) axis [19]. Restriction of dietary isoleucine is enough to attenuate western diet-induced (WD) obese-associated hepatic steatosis by reducing hepatic lipid deposition and smaller LD [19]. In addition, reduction of dietary isoleucine is enough to promote the metabolic health of young and old HET3 mice, improving leanness and glycemic control in both sexes, reducing frailty, extending the lifespan, and remodeling hepatic metabolism in a sex-dependent manner [20]. However, a high-protein diet that is enriched in BCAA, is presently the most useful therapeutic for NAFLD which leads to a reduction of liver fat by 36%–48 % in NAFLD patients within 6 weeks [[21], [22], [23]]. In particular, high protein diet enriched in BCAA has been widely used to treat hepatic steatosis-related liver disease in both preclinical and clinical trials [[24], [25], [26]]. Recent research also found that dietary leucine and isoleucine ameliorate steatosis by promoting polyubiquitination of PLIN2 via powerfully binding to and activating UBR1, targeting the degradation of PLIN2 [27]. Leucine and isoleucine-induced activation of UBR1/2 ubiquitination is necessary to ameliorate hepatic steatosis and obesity induced by high-fat diet (HFD) [27]. Nevertheless, these contradictory findings uncover the complex role of BCAA in the pathogenesis and progress of NAFLD.

Fatty liver hemorrhagic syndrome (FLHS) induced by NAFLD in poultry, notably laying hens, has been a giant cause of death in commercial caged laying hens [28]. FLHS is characterized by the accumulation of large amounts of hepatic lipid and abdominal fat and accompanied by hepatic hemorrhage [29]. The pathogenesis of FLHS is similar to NAFLD in humans, resulting in considerable mortality of laying hens during and after the laying peak period due to liver rupture leading to internal bleeding [29]. It is worth noting that, until now, our knowledge about the impacts of BCAA on the pathogenesis of NAFLD was primarily based on either a single dietary BCAA restriction or intake of all three BCAA at the same time. However, the metabolic impacts of dietary BCAA supplementation on laying hens under the pathological circumstances of FLHS remains unknown. Therefore, an overall analysis of the functions of dietary supplementation with BCAA on NAFLD and FLHS, applying to laying hen models that partly imitate the human NAFLD and appropriate dosing, is urgent to be solved. Given the load of NAFLD on humans and FLHS on laying hens, lack of effective therapies, and contradictory reports relating dietary BCAA intake to NAFLD progress, there is a strong reason to better comprehend the impacts of dietary BCAA supplementation on NAFLD, which can find effective therapeutics. Here, we attempt to detect whether and how high dietary BCAA supplementation contributes to NAFLD development by performing transcriptome, proteome, and metabolome in the serum and liver of middle-aged laying hens with NAFLD. We suggest that supplementation of 67 % three High BCAA (a maximum tolerance dose), but not leucine, isoleucine, and leucine plus isoleucine, is both required and enough to ameliorate NAFLD by reprogramming liver metabolism. These data indicate that dietary High BCAA is a useful treatment for FLHS in laying hens or potentially human NAFLD and recommend that a modest increase of dietary BCAA may be a convertible way to ameliorate and treat NAFLD and FLHS.

## 2 Materials and methods

### 2.1 Diets, birds, and experimental management

Corn and soybean meals are major ingredients of a corn-soybean-type basal diet and are made according to NRC (1994) [30] and China National Feeding Standard of Chicken (NY/T33-2004) [31]. To determine the requirement of dietary BCAA in laying hens during the laying peak period, we collected the published research references related to the requirement and excess of dietary BCAA. These reports most commonly evaluated the impacts of different dietary BCAA levels on productive performance and egg quality, but did not include other metabolic parameters such as lipid metabolic homeostasis (Table S1). These data suggest that dietary isoleucine and valine display adverse impacts on body weight and productive performance when their levels are higher than 1.00–1.15 % and 1.09–1.36 % (about over than the control diet 67 %), respectively (Table S1). There is no adverse report in terms of dietary leucine excess and also have not been investigated. Here, we design an amino acid-determined control (Ctrl AA) diet including eight usual amino acids (i.e., methionine, lysine, threonine, tryptophan, arginine, leucine, isoleucine and valine); the dietary amino acid composition reflects that of a conventional laying hen diet in which a fixed, isocaloric and iso-proteinic macronutrient background that are derived from corn and plant-based protein. This Ctrl AA diet contains optimal BCAA requirements that include leucine (1.20 %), isoleucine (0.65 %) and valine (0.70 %) based on NRC (1994) [30], China National Feeding Standard of Chicken (NY/T33-2004) [31] and our previous reports [[32], [33], [34]]. To analyze the distinct role of each and combined BCAA but not include valine because we have evaluated the adverse impact of high valine on NAFLD in this model [[32], [33], [34]]. We generated a diet series based on this Ctrl AA diet and the manipulations of BCAA levels were performed so that the in which leucine (High Leu), the isoleucine (High Ile), the leucine and isoleucine (High LeuIle), or the three BCAA (High BCAA) were increased by 67 % (Table S2). In addition to BCAA, the levels of other amino acids, protein and energy in the basal diet were adjusted to be consistent with each group.

A total of 180 Fengda No.1 laying hens, aged 42 weeks and in good health, were selected based on similar body weight and laying rate. Laying hens were randomly assigned to 5 experimental groups, with each group comprising 6 replicates of 6 laying hens (6 birds per cage). The experiment spanned 9 weeks, comprising a one-week pre-feed period and an 8-week experimental period. All hens were housed in a climate-controlled room, with the temperature maintained at approximately 23 °C. Throughout the experiment, laying hens were exposed to a 16h photoperiod using artificial lighting. Laying hens were given water and drink freely and fed a complete feed mixture diet weighing approximately 100g twice daily.

### 2.2 Sample collection and processing

A total of 60 hens were randomly selected and fasted for 12h at the end of the 8-week experiment, with 2 hens per replication and 6 replications per group. A wing vein blood sample was collected and separated by centrifugation at 3000×*g* for 10min and stored at −80 °C. The sample tissues were collected after hens were euthanized with pentobarbital sodium and sacrificed.

### 2.3 Serum and liver biochemical parameter analysis

Hepatic tissues were homogenized in the ratio of 1:4 or 1:9 (w/v) using 0.9 % sterile normal saline on ice and centrifugation at 4 °C 3500×*g* for 15min. Total protein was detected using a BCA protein assay kit (Nanjing Jiancheng Bioengineering Institute, Nanjing, China) and stored at −80 °C. Triglyceride (TG), NAD^+^/NADH, total cholesterol (T-CHO), catalase (CAT), lipase (LPS), reduced glutathione (GSH), trypsin, oxidized glutathione (GSSG), high-density lipoprotein cholesterol (HDL-C), total glutathione (T-GSH), total amino acid (TAA), malondialdehyde (MDA), total bile acid (TBA), total antioxidative capacity (T-AOC), alanine aminotransferase (ALT), total lipases (TL), total superoxide dismutase (T-SOD), aspartate aminotransferase (AST), lipoprteinlipase (LPL), gamma-glutamyltransferase (GGT), NADP^+^/NADPH, non-esterified fatty acid (NEFA), adenosine triphosphate (ATP), low-density lipoprotein cholesterol (LDL-C), hepaticlipase (HL), glutathione peroxidase (GSH-Px), α-amylase (AMS), glucose (Glu) and glycogen (Gly) were analyzed in fasted-laying hens by commercial kits (Nanjing Jiancheng Bioengineering Institute, Nanjing, China), respectively, according to the manufacturer's protocol. Briefly, a BeadBeater™ tissue grinder (Biospec MiniBeadbeater 16, USA) was used for hepatic tissue homogenization and the optical density (OD) value was detected by spectrophotometer (UV-1601 UV-VIS Spectrophotometer, Shimadzu Corporation, Tokyo, Japan).

### 2.4 ELISA analysis

The determination of fatty acid synthase (FASN), acetyl coenzyme A (acetyl-CoA), interleukin (IL-6), acetyl CoA carboxylase (ACC), glucagon (GC), IL-17, ATP citrate lyase (ACLY), insulin, very-low-density lipoprotein cholesterol (VLDL), hormones estradiol (E2), monoacylglycerol lipase (MGL), hormone fibroblast growth factor 19 (FGF19), follicle-stimulating hormone (FSH), tumor necrosis factor-α (TNF-α), adipose triglyceride lipase (ATGL), luteinizing hormone (LH), IL-1β, hormone-sensitive triglyceride lipase (HSL), and interferon-γ (IFN-γ) in the liver and fat of fasted-laying hens were by chicken ELISA kits (Nanjing Jiancheng Bioengineering Institute, Nanjing, China). All analysis was conducted according to the kit instructions. These hepatic and fat tissues were homogenized using a BeadBeater™ tissue grinder (Biospec MiniBeadbeater 16, USA) and the OD value was analyzed by spectrophotometer (UV-1601 UV-VIS Spectrophotometer, Shimadzu Corporation, Tokyo, Japan).

### 2.5 Histological analysis

Liver tissues were either stored at 10 % paraformaldehyde, 2.5 % glutaraldehyde or embedded in the Tissue-Tek OCT compound. The hepatic tissue slides were stained with hematoxylin and eosin (H&E), picrosirius red and Masson to analyze lipid deposition, liver injury and fibrosis, respectively. The frozen samples were sectioned and stained with Oil Red O to observe lipid deposition and LD. Images were obtained by Nikon 90i Eclipse upright microscope (Nikon Eclipse 80i, Nikon, Tokyo, Japan).

### 2.6 Transmission electron microscope (TEM) assay

Hepatic tissues were first fixed by 2.5 % glutaraldehyde for 4h and then subjected to 1 % osmium tetroxide (OsO_4_) for 2h at room temperature (RT) avoiding illumination. Exhausted fixing agents were removed by 0.1 M pH7.0 phosphate buffer 3 times and each 15min. Samples were further dehydrated by graded ethanol series (50 %, 70 %, 80 %, 90 %, 95 % and 100 %) and pure acetone for each 20min at RT. The specimen was fixed in a mixture (1:1) of pure acetone and Epon 812 resin (SPI Supplies Inc. PA, USA) for 1h at RT, and then transferred to a mixture (1:3) of pure acetone and Epon 812 resin for 3h and finally to Epon 812 resin for overnight. The specimen was transferred to an Eppendorf containing Epon 812 resin and heated at 70 °C for 9h. The Specimen was cut by a Leica UC 7 microtome (Leica, Vienna, Austria) with a diamond knife (Diatome, Switzerland) to obtain ultra-thin sections (70 nm). Sample sections were stained by uranyl acetate and alkaline lead citrate for 10min, respectively, and observed at 80 kV of accelerating voltage using an H7650 TEM (Hitachi, Ibaraki, Japan). Images were acquired by a Gatan 830 CCD camera (Gatan, CA, USA). Hepatic cellular ultrastructure, LD droplet, peroxisome, mitochondria, and autophagic vacuoles were quantified in at least 12 high-power fields (covering 100 μm^2^) (n = 3 hens per group). All TEM analysis was conducted in the Bio-ultrastructure Analysis Lab of the Analysis Center of Agrobiology and Environmental Sciences, Zhejiang University.

### 2.7 Immunofluorescence analysis

The fresh frozen sections were rewarmed at RT to remove moisture and the tissue self-fluorescence was quenched by using a self-fluorescence quencher and washed with PBS (pH7.4). Reactive oxygen species (ROS) dye solution was added and incubated at 37 °C to dyeing and washed with PBS (pH7.4). DAPI dye solution was added and incubated at RT for DAPI re-staining nuclei and washed with PBS (pH7.4), and anti-fluorescence quenching sealing tablets to sealing. The image was acquired using a Leica Sp8 laser scanning confocal microscope and analyzed by ImageJ.

### 2.8 Real-time quantitative PCR (RT-qPCR)

The mRNA expression was analyzed by real-time quantitative PCR (RT-qPCR). Total RNA was extracted by TRIzol reagent (Vazyme Biotechnology, Nanjing, Jiangsu, China) and the quality and quantity were detected by a NanoDrop 2000 spectrophotometer (Thermo Fisher Scientific, Massachusetts, USA). cDNA was synthesized using a HiScriptIIqRT SuperMix Reverse Transcriptase (Vazyme Biotechnology, Nanjing, Jiangsu, China). RT-qPCR was performed using an SYBR Premix PCR kit (Vazyme Biotechnology, Nanjing, Jiangsu, China) by the CFX96TM Real-Time System (Bio-Rad, Hercules, CA, USA). Primers were used for RT-qPCR and β-actin was used as a reference gene (Table S3). Each sample was run in triplicate, and the 2^−ΔΔCt^ method was used to calculate the relative mRNA expression of the target gene.

### 2.9 Western blot assay

Hepatic tissues were washed with ice-cold PBS and homogenized with ice RIPA buffer containing 1 mM Phenylmethanesulfonylfluoride (PMSF), 0.5 mM protease inhibitor, and 0.5 mM phosphatase inhibitor and then reaction at ice for 20min (Beijing Solarbio Science & Technology Co., Ltd.). Whole-tissue lysates were collected by centrifugation and protein concentration was quantified using a BCA protein assay kit (Nanjing Jiancheng Bioengineering Institute, Nanjing, China). Samples were subjected to 4–20 % sodium dodecyl sulfate-polyacrylamide gel electrophoresis (SDS-PAGE) and transferred to polyvinylidene difluoride (PVDF) membranes (Millipore, MA, USA) using semi-dry transfer system. The membranes were blocked by TBST containing 5 % non-fat dried milk for 2h at RT and then incubated with primary antibodies overnight at 4 °C. After incubation with HRP-conjugated secondary antibodies for 1h, immunoreactive proteins were analyzed by an ECL detection system (Millipore).

Proteins were assessed with the following primary antibodies (Abs). Abs to anti-GCN2 (ET1704-68), anti-ATG5 (ET1611-38), anti-eIF2α (HA500385), anti-p-eIF2α (ET1603-14), anti-ATF4 (ET1612-37), anti-ATG7 (ET1610-53), anti-LC3I (ER1802-2), anti-LC3II (ET1701-65), anti-4EBP1 (ER1706-64), anti-p-4EBP1/2/3 (Thr45, RT1004), anti-p70-S6K1 (ER3125, HUABIO), anti-p-p70-S6K1 (ET1608-53), anti-S6 (HA600081), anti-p-S6 (S235 + S236, HA721275), anti-CHOP (ET1703-05), anti-HNF4A (ET1611-43), anti-GRP78 (HA601076), anti-AHR (ET1703-11) and anti-FASN (ET1701-91) were provided by Hangzhou HuaAn Biotechnology Co., Ltd. (Hangzhou, China). anti-mTOR (Cat No. 28273-1-AP) and anti-p-mTOR (Cat No. 67778-1-Ig) were provided by Proteintech Group, Inc. (Wuhan, China). anti-ERK (Cat No. #4695), anti-p-ERK (Cat No. #5726), anti-AMPKα (Cat No. #2532), anti-p38-MAPK (Cat No. #4511), anti-p-p38-MAPK (Cat No. #4511) were purchased from Cell Signaling Technology (CST, BS, USA). The protein expression was normalized using the anti-β-actin antibody (42 kDa, M1210-2) purchased from Hangzhou HuaAn Biotechnology Co., Ltd (Hangzhou, China).

### 2.10 Analysis of serum free amino acids

Serum was deproteinized by a sulfonic acid (5 %) (V: V = 1:1), vortexed 30s and centrifuged at 18,000×*g* for 30min. Subsequently, a 20 μl aliquot of the supernatant was injected into a high-performance liquid chromatography (HPLC) column (Hitachi L-8900 Amino Acid Analyzer, Hitachi High Technologies Japan, Inc. Tokyo, Japan). Amino acids were separated by cation exchange using lithium buffers, with the UV light detection (570 nm) of individual amino acids (440 nm for proline) conducted by post-column ninhydrin derivatization.

### 2.11 Targeted metabolomic analysis

Quantification of serum tryptophan metabolites and hepatic fatty acids was performed by an ultra-high-performance liquid chromatography-mass spectrometry (UHPLC-MS) system comprising a 1290 UHPLC system (Agilent) coupled to a QTRAP 6500^+^ Mass Spectrometer (AB Sciex). Serum samples (100 μl) were prepared by protein precipitation with the addition of ice methanol (500 μl) and 10 μl 200 ng/ml containing mixed stable isotope labeled internal standards (l-tryptophan-D_5_ (Sigma-Aldrich)). Samples were vortex mixing 60s and placed at −20 °C for 1h to precipitate protein and then centrifugation (14,000×*g*, 20min, 4 °C). The hepatic samples (100 μg) were homogenized with 300 μl of isopropanol/acetonitrile (1:1) which contained mixed internal standards and centrifugation (12,000×*g*, 10min, 4 °C). The supernatants (5 μl of serum and 10 μl of liver) were injected directly into a waters ACQUITY UPLC BEH C18 column (2.1 × 100 mm, 1.7 μm) for the LC-MS/MS system analysis. Raw data (Analyst Software v.1.6.2; AB Sciex) was imported into the MultiQuant v.3.0 analysis software for the integration of Q1/Q3 peaks using multiple reaction monitoring, as well as for data normalization.

### 2.12 Untargeted metabolomic analysis

Untargeted metabolomic analysis of liver was conducted based on a UHPLC-MS system comprising a 1290 UHPLC system (Agilent) coupled to a Q Exactive HF-X Mass Spectrometer (AB Sciex). The hepatic tissues (50 mg) were homogenized with 400 μl of methanol/water (4:1) which contained mixed internal standards (0.02 mg/ml, 2-chloro-l-phenylalanine, Adamas-beta). Samples were ultrasonic extracted at 5 °C for 30min and placed at −20 °C for 30min, and then centrifugation (13,000×*g*, 10min, 4 °C). The supernatants (3 μl) were directly injected into a waters ACQUITY UPLC HSS T3 column (100 mm × 2.1 mm, 1.8 μm) for the LC-MS/MS system analysis. The analysis of Raw data (Analyst Software v.1.6.2; AB Sciex) was imported into the ProgenesisQI analysis software. The metabolomic sequencing of the liver was performed by Shanghai Majorbio Bio-pharm Technology Co., Ltd. and data analysis was based on the free online platform of Majorbio Cloud Platform (https://cloud.majorbio.com/). (Shanghai, China).

### 2.13 RNA-sequencing and data analysis

Hepatic total RNA was extracted by TRIzol reagent (Takara code: 9109, Shiga, Japan) and the quality and quantity were quantified by a Nanodrop 2000 spectrophotometer (Thermo Fisher Scientific, Massachusetts, USA). RNA integrity numbers (RINs) were determined by Agilent2100 and the total RNA was subjected to mRNA polyA purification. The mRNA was fragmented using a fragmentation buffer and copied into first-strand cDNA by random hexamers. cDNA was end-repaired and dA-Tailing, and then purified and enriched by PCR and checked for quality and quantity by qPCR. The final products were paired-end sequenced using a NovaSeq 6000 Sequencing System (Illumina) and the RNA sequencing raw data was processed and obtained clean sequences and then mapped to the chicken genome (Gallus_gallus, GCF_016700215.2, https://ftp.ncbi.nlm.nih.gov/genomes/refseq/vertebrate_other/Gallus_gallus/latest_assembly_versions/GCF_016700215.2_bGalGal1.pat.whiteleghornlayer.GRCg7w/) using HISAT2 v.2.1.0.13. We considered differentially expressed genes (DEGs) with adjusted *p*-values less than 0.05 and absolute fold change larger than 1 as significant DEGs. The RNA Seq was performed by Shanghai Majorbio Bio-pharm Technology Co., Ltd. and data analysis was based on the free online platform of Majorbio Cloud Platform (https://cloud.majorbio.com/) (Shanghai, China).

### 2.14 Ubiquitin-modified proteome analysis

Data independent acquisition (DIA) quantitative proteomics was used for the analysis of ubiquitinating proteome. Briefly, total protein was extracted and quantified concentrations using a BCA protein assay kit (Bio-Rad) from the whole-tissue lysates and then subjected to 4–20 % SDS-PAGE. Sample protein was reduced with 100 mM DTT for 5min at 95 °C and cooled to RT and then addition of 200 μl UA buffer (8 M Urea, 150 mM Tris-HCl, pH8.0) for mixed and centrifugation (12,000×*g*, 15min, RT), repeat once. Addition of 100 μl IAA (50 mM IAA in UA) for oscillation (1min, 600×*g*) and avoid light at RT for 30min and then centrifugation (12,000×*g*, 10min, RT). 100 μl UA buffer was added for centrifugation (12,000×*g*, 10min, RT) and repeat twice, and then addition of 100 μl NH_4_HCO_3_ buffer for centrifugation (14,000×*g*, 10min, RT) and repeat twice. Addition of 40 μl trypsin buffer (6 μg trypsin in 40 μl NH_4_HCO_3_ buffer) for oscillation (600×*g*, 1min) and stay at 37 °C for 16–18h and then centrifugation (12,000×*g*, 10min, RT). Peptides were dissolved using 0.1%TFA buffer and desalted by Waters 500 mg C18 Cartridge (WAT036945) and then frozen and lyophilized. Peptides were re-dissolved in 0.1%FA buffer and quantified concentrations using Nanodrop 2000.

Peptides were frozen and lyophilized and the Cell Signaling Technologies PTMScan Ubiquitin Remnant Motif (K-ε-GG) Kit (CST #5562) was used to isolate the ubiquitin remnant-containing peptides. Lyophilized peptides were dissolved in 10 μl 0.1 % FA buffer for MS analysis. LC-MS analysis was analyzed on a UHPLC-MS system comprising a Vanquish Neo UHPLC system (Waters) coupled to an Orbitrap Astral Mass Spectrometer (Thermo Scientific) and an EASY C18 column (75μm × 120 mm, 3 μm). Samples were directly injected into a waters Trap C18 Column (100μm × 20 mm, 5 μm) and then into μPAC Neo High Throughput column for the LC-MS/MS system analysis. Mass spectra were acquired in DIA mode and the resulting high-quality reads were analyzed using Spectronaut (version 18, Biognosys AG) and then mapped to the chicken reference proteome https://www.uniprot.org/9031 uniprot-gallus (Chicken) [9031]-51562-20231,230.fasta. The MS analysis was conducted by Shanghai Bioprofile Technology Company, Ltd. (Shanghai, China).

### 2.15 Mass spectrometry analysis

Hepatic whole-tissue lysates were boiled into 1 × SDS loading buffer for destained, reduced and alkylated and then digested with trypsin at 95 °C for 20h. The peptides were desalted, frozen and lyophilized and then re-dissolved by 0.1%FA buffer and stored at −20 °C. LC-MS analysis was analyzed on a UHPLC-MS system comprising an Easy nLC 1000 system (Thermo Fisher) coupled with a Q Exactive Mass Spectrometer (Thermo Fisher) and an EASY-Spray C18 column (15cm × 75 mm, 3 mm). The resulting MS/MS raw file was processed by Proteome Discoverer 2.5 (Thermo Fisher) and then mapped to the chicken reference proteome https://www.uniprot.org/9031 uniprot-gallus (Chicken) [9031]-51562-20231,230.fasta. The MS analysis was conducted by Shanghai Bioprofile Technology Company, Ltd. (Shanghai, China).

### 2.16 Statistical analysis

Unless otherwise stated, the data variance analysis was conducted by the homogeneity of variance test (SPSS 23.0). Statistical analysis between five groups was done using one-way ANOVA followed by a Tukey-HSD multiple comparison test (SPSS 23.0), which means that different lower letters are statistically significant. Data analysis between the two groups was done using an unpaired Student's t-test with Welch's correction (SPSS 23.0). Results are presented as mean ± standard error (SE). The Spearman correlation analysis was performed using the LC-Bio Cloud Platform with an R package (version 3.6.3) (https://www.omicstudio.cn/). We considered a significant difference at *p* < 0.05 and/or ∗ represents *p* < 0.05, ∗∗ represents *p* < 0.01 and ∗∗∗ represents *p* < 0.001. Graphs were generated by GraphPad Prism 8.0 software (GraphPad Software, San Diego, CA, USA).

## 3 Results

### 3.1 High BCAA diet reprograms hepatic BCAA metabolism

**Fig. 1:**
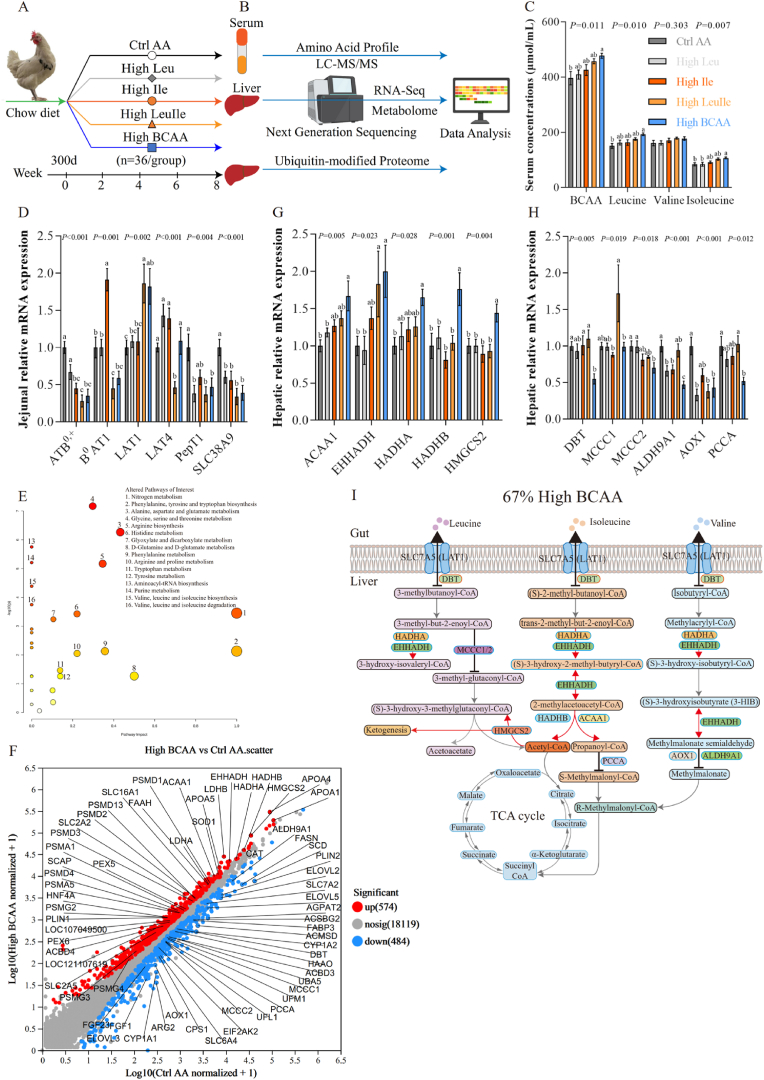
High BCAA diet reprograms hepatic BCAA metabolism (n = 5–6/group). (A) Experimental scheme (n = 36/group). (B) Workflow for multi-omics. (C) Serum concentrations of BCAA. (D) mRNA expression of amino acid transporters in jejunum. (E) Representation of pathways altered in Ctrl AA and High BCAA-fed laying hens based on serum amino acid metabolomics data. Metabolic pathways of interest are highlighted. (F) Volcano plot of hepatic DEGs in the transcriptomic analysis of Ctrl AA and High BCAA-fed laying hens. DEGs of interest are visualized. (G–H) Hepatic mRNA expression of DEGs of BCAA metabolism in Ctrl AA, High Leu, High Ile, High LeuIle or High BCAA-fed laying hens. (I) Schematic of reprogramming hepatic BCAA metabolism mediated by 67 % High BCAA-feeding. Red line: leads to activation. Black line: leads to inhibition. Gray line: not impact.

In an initial study, middle-aged Fengda No.1 laying hens (over 300 days old) during the late laying peak were selected as a NAFLD model, as they can spontaneously develop FLHS under a high-energy, high-protein diet, with pathogenesis similar to human NAFLD [28,29]. Fengda No.1 laying hens are bred by three strains (R1, D1 and D3 strain) and have been extensively used in our recent NAFLD studies because they better mirror the genetic diversity of the human population than any single hybrid strain (Fig. 1A) [33,34]. Firstly, we analyzed the effects of dietary intake BCAA on body weight phenotype and found consumption of either the High Leu, High Ile, High LeuIle or High BCAA diet did not affect average body weight and body weight gain during feeding (Figs. S1A and B). Furthermore, serum free amino acids were determined using targeted metabolomic (Fig. 1B) and found that High BCAA feeding dramatically increased serum leucine, isoleucine and total BCAA, but did not include valine although it displayed elevated (Fig. 1C). We further analyzed the effect of High BCAA on intestinal amino acid absorption and transportation. Hens fed a High LeuIle or High BCAA diet specifically upregulated the BCAA preferred transporter L-type large neutral amino acid transporter 1 (LAT1) mRNA expression level in the jejunum (Fig. 1D). But, did not affect other neutral amino acid transporters such as ATB^0,+^, B^0^AT1, LAT4, PepT1 and SLC38A9 or other intestinal segments (Figs. S1C and D). The elevation of serum BCAA was not mediated by the inhibition of BCAA catabolism in the peripheral tissues such as the pectoral muscle because the BCAA is first catabolized in most peripheral tissues, rather than in the liver. qPCR confirmed that hens fed either the High Leu, High Ile, High LeuIle or High BCAA did not impact the mRNA expression of BCAA transportation and catabolism-associated genes in pectoral muscle, in addition to significantly downregulated PPM1K (Figs. S1E–G). Spearman correlation analysis was used to coordinate the mRNA expression of intestinal amino acid transporters and serum free amino acids data and identified the absorption and transportation of BCAA is mainly mediated by intestinal LAT1 (Figs. S1H and I). Metaboanalyst 5.0 was performed to coordinate the changed amino acid data and identify pathways altered in response to High BCAA intervention (Fig. 1E). We found alterations in several central amino acid metabolic processes, including nitrogen metabolism, AAA biosynthesis and metabolism, glycine, serine and threonine metabolism, alanine, aspartate and glutamate metabolism, arginine biosynthesis, histidine metabolism, and BCAA biosynthesis and degradation. Given both the increased circulating BCAA and unchanged BCAA catabolism in the peripheral tissues, we conclude that High BCAA feeding promotes intestinal LAT1-mediated BCAA uptake and mainly affects liver BCAA metabolism.

To investigate whether and how High BCAA feeding influences hepatic BCAA metabolism, we conducted RNA sequencing (RNA-seq) in the liver of fed Ctrl AA and High BCAA diet hens (Fig. 1B). We observed a profound effect of a High BCAA diet on hepatic transcription profile, with 574 genes upregulated significantly and 484 genes downregulated significantly in High BCAA diet-fed hens, respectively (Fig. 1F). Differentially expressed genes (DEGs) analysis revealed that eleven genes linked to BCAA metabolism, DBT, MCCC1, MCCC2, ALDH9A1, AOX1 and PCCA are downregulated, ACAA1, EHHADH, HADHA, HADHB and HMGCS2 are upregulated*,* respectively, as evidenced by qPCR (Fig. 1G and H). These downregulated BCAA catabolism-associated genes are mainly linked with the degradation of branched-chain α-ketoacids (BCKAs) and the BCAA catabolism final products of propionyl-CoA and succinyl-CoA. Yet, these upregulated BCAA catabolism associated genes primarily in response to the metabolism of acetyl-CoA and 3-hydroxyisobutyrate (3-HIB), indicative of increased ketogenesis, probably in response to increased acetyl-CoA (Fig. 1I). Kyoto Encyclopedia of Genes and Genomes (KEGG) enrichment analysis revealed that DEGs changed by High BCAA intake were enriched in valine, leucine and isoleucine degradation (Fig. S1J). Notably, these significantly upregulated DEGs including ACAA1, EHHADH, HADHA, HADHB and HMGCS2 not only participate in 10.13039/100022047BCAA catabolism but are also involved in fatty acid β-oxidation and ketogenesis, which is supported by upregulated ketone body transporter SLC16A1 (Fig. 1F) and decreased hepatic and circulating acetyl-CoA (discuss in later). This likely reflects altered hepatic lipid metabolism, because hepatic BCAA catabolites are not used for DNL but turn to β-oxidation and ketogenesis. Together, our analyses indicate profound changes in hepatic metabolism upon High BCAA feeding, in particular, hepatic BCAA metabolism.

Performing mass spectrometry (MS) analysis, we further identified 36 relatively altered proteins in the liver of fed Ctrl AA and High BCAA diet hens. The abundance of these proteins encoded by these genes involved in the degradation of BCAA and the catabolism of final products, respectively (Figs. S2A–F). Intriguingly, the abundance of proteins encoded by the branched-chain α-keto acid dehydrogenase (BCKDH) complex (BCKDC), including DBT, BCKDHB and BCAT1, were partly higher in High BCAA-fed hens (Figs. S2A and C). We examined the possibility that these hens only have a branched-chain amino acid transaminase 1 (BCAT1), which mediated BCKAs reamination and the formation of a mitochondrial BCAA metabolon involving BCAT1 and BCKDH in controlling BCAA catabolism [35]. These results suggest that inhibition of the degradation of BCKAs leads to the accumulation of serum BCAA and the final products of acetyl-CoA, propionyl-CoA and succinyl-CoA do not into the TCA cycle and turn into ketogenesis. Together, we suggest that a High BCAA diet was enough to induce profound changes in hepatic BCAA metabolism in middle-aged laying hens; this is probably due to the effects of amino acid interaction and metabolic reprogramming.

### 3.2 High BCAA diet downregulates tryptophan metabolism and leads to indole-3-lactic acid depletion

**Fig. 2:**
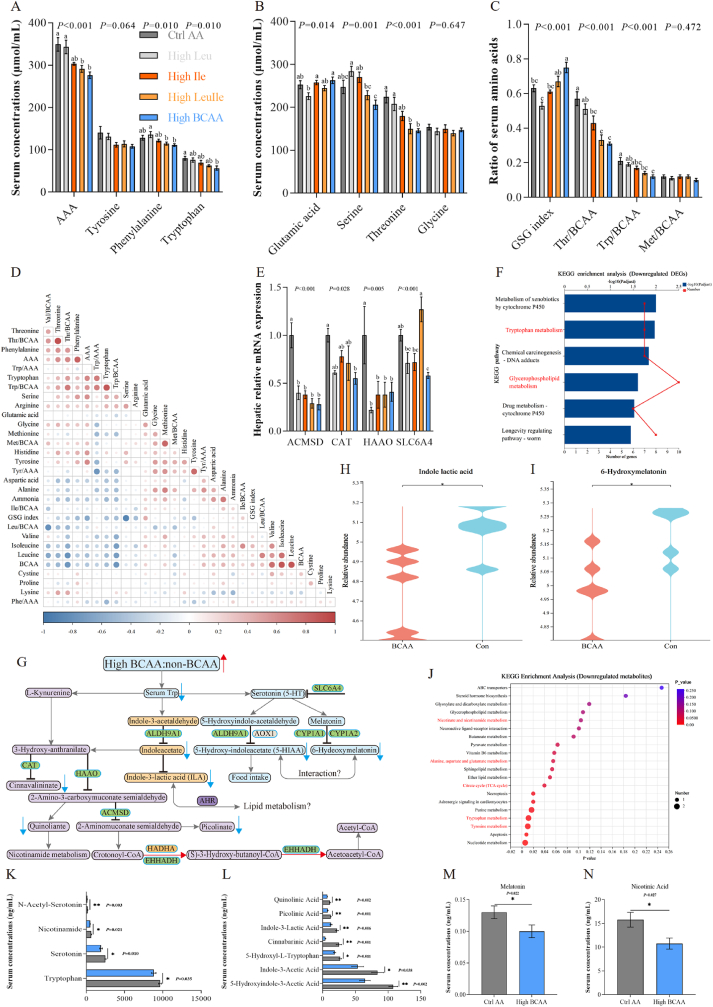
High BCAA diet downregulates tryptophan metabolism and leads to indole-3-lactic acid depletion (n = 5–6/group). (A–C) Serum amino acid concentrations and amino acid ratio. (D) Spearman's correlation of serum free amino acids involved in BCAA and AAA metabolism in laying hens. (E) Hepatic mRNA expression involved in the tryptophan metabolism. (F) KEGG enrichment analysis based on downregulated DEGs in Ctrl AA and High BCAA groups. (G) Schematic of tryptophan metabolic pathway. Red line: leads to activation. Black line: leads to inhibition. Blue line: leads to a decrease. Gray line: not impact. (H–I) Hepatic tryptophan metabolites abundances from LC-MS/MS in Ctrl AA and High BCAA groups. H, Indole lactic acid. I, 6-Hydroxymelatonin. (J) KEGG enrichment analysis based on downregulated metabolites from Ctrl AA and High BCAA groups. (K–N) The serum metabolites of tryptophan from Ctrl AA and High BCAA groups.

How hepatic BCAA metabolic reprogramming induced by High BCAA feeding affects host metabolic homeostasis, amino acid metabolism, specifically, has not been investigated. Long-term intake of high BCAA diets results in amino acid imbalance, in particular a change in the relative quantity of BCAA and other amino acids, particularly tryptophan and threonine [9]. To determine whether and how High BCAA feeding impacts amino acid metabolism, we next analyzed the impacts of long-term intake of High BCAA diet on serum tryptophan and threonine. Consistently, serum tryptophan, total AAA and threonine were significantly reduced by an eight-week High BCAA intervention, but not either the High Leu, High Ile or High LeuIle, revealing intake of more BCAA influenced host amino acid homeostasis (Fig. 2A and B). The glutamate-serine-glycine (GSG) index [7], a possible biomarker of NAFLD that is independent of body mass index (BMI) and reflects host amino acid homeostasis, was significantly increased after High BCAA intervention (Fig. 2C). Intake of either the High Leu, High Ile, High LeuIle, or High BCAA had no significant effect on other essential amino acids (EAA) and non-EAA (Figs. S3A and B). To analyze whether there are other non-BCAA that mediated this impact, the level of each EAA was calculated between treatment diets and was maintained consistently across diet treatments (Table S2). We next tested the results that there is a change in the relative quantity of serum BCAA and other amino acids, in which the ratio of tryptophan and threonine relative to serum total BCAA were dramatically decreased by High BCAA intervention but had no impact on single BCAA relative to serum total BCAA or single AAA relative to serum total AAA (Fig. 2C and Fig. S3C). The reduction of serum tryptophan and threonine was not mediated by dietary intake although we observed the lowest feed intake in High BCAA intervention treatment (Figs. S3D and E). When dietary BCAA intake was plotted (Figs. S3F and G), the intake of non-BCAA among all groups was maintained at a consistent level and no additional supplementation, suggesting modulation of intake of three BCAA to a target intake (Table S2). In fact, the inverse relationship between BCAA and tryptophan, which contend for peripheral and central transportation by the LAT1 is also a response in the ratio of tryptophan/BCAA in the serum and diet [20,36], where High BCAA-fed laying hens exhibit the lowest serum and dietary tryptophan/BCAA ratio (Fig. 2C and Fig. S3H). Spearman's correlation further clarified the interplay between High BCAA intervention and serum free amino acid that the changes in tryptophan, threonine, ratio of tryptophan and threonine relative to total BCAA were negatively correlated with elevated serum total BCAA (Fig. 2D and Fig. S3I). These results suggest that tryptophan and threonine are prioritized and regulated, affecting amino acid homeostasis induced by the interaction between BCAA and these amino acids.

To examine how dietary High BCAA intervention affects tryptophan and threonine signaling, we performed RNA-seq followed by qPCR validation. We found DEGs involved in tryptophan metabolism were significantly downregulated after High BCAA intervention (Fig. 1F), as evidenced by qPCR and KEGG enrichment analysis (Fig. 2E and F and Fig. S1J). RNA-seq uncovered significantly downregulated tryptophan metabolism mediated by High BCAA intervention mainly involved in the metabolism of kynurenine (Kyn), 5-hydroxytryptamine (also refers to serotonin, 5-HT), and indole pathways (Fig. 2G) [37,38]. Liver untargeted metabolomics demonstrated significantly decreased tryptophan metabolites such as indole lactic acid (ILA), 6-hydroxymelatonin, 5-methoxyindoleacetate and indole carboxylic acid sulfate (Fig. 2H and I and Figs. S3J and K), which is further confirmed by inhibited tryptophan metabolism based on downregulated metabolites (Fig. 2J). Serum targeted metabolomics showed that tryptophan and its metabolites, including 5-HT, nicotinamide, N-acetyl-serotonin, 5-hydroxyindole-3-acetic acid (5-HIAA), indole-3-acetic acid, 5-hydroxyl-l-tryptophan, cinnabarinic acid, ILA, picolinic acid, quinolinic acid, melatonin, and nicotinic acid were deceased by High BCAA intervention (Fig. 2K-N and Fig. S3L). As a monoamine neurotransmitter that controls appetite, the downregulation of tryptophan metabolism is of the essence in feeding behavior because tryptophan is the only precursor for 5-HT production [9]. However, we found High BCAA intervention did not affect average feed intake (Figs. S3D and E), which can be explained by dietary background differences (i.e. a high-carbohydrate, low-fat diet background and isocaloric and iso-proteinic macronutrient background). Reduction of serum tryptophan, 5-HT and its main metabolites 5-HIAA and melatonin mediated by High BCAA intake may reflect the limitation of peripheral tryptophan absorption (Fig. 1D) and the inhibition of hepatic 5-HT uptake mediated by 5-HT transporter SLC6A4 (Fig. 2E). Spearman's correlation analysis was performed to coordinate the serum amino acid profile and tryptophan metabolites data and identified 5-HT, nicotinamide, N-acetyl-serotonin, 5-HIAA and picolinic acid were negatively altered in response to elevated serum BCAA induced by High BCAA feeding while positively in response to declined circulating tryptophan (Figs. S3M and N). Surprisingly, one of these significantly declined hepatic and circulating tryptophan metabolites was ILA (Fig. 2H and L), which was recently identified as positively associated with T2D risk and promoted obesity [39,40]. This likely reflects High BCAA-mediated tryptophan ligand ILA depletion may induce lipid metabolism remodeling. Together, these results unveil that long-term High BCAA intervention is sufficient to induce amino acid imbalance and in particular for High BCAA feeding to maximally influence tryptophan-mediated ligand ILA depletion.

### 3.3 High BCAA diet alleviates hepatic steatosis and reprograms lipidome

**Fig. 3:**
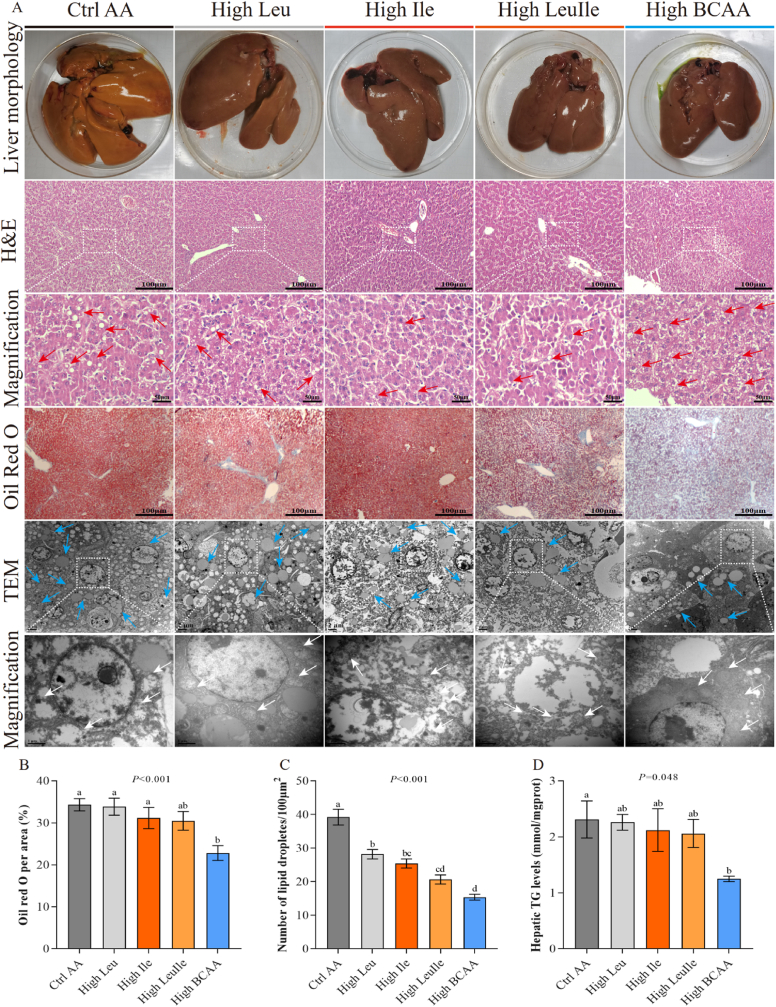
High BCAA diet alleviates hepatic steatosis (n = 5–6/group). (A) Morphology phenotype, H&E staining, Oil Red O staining, and TEM of livers from hens fed with High Leu, High Ile, High LeuIle or High BCAA diets (n = 3/group). (B) Oil Red O staining area (n = 3/group). (C) Hepatic LD numbers from TEM (n = 3/group). (D) Hepatic TG levels.

It has been revealed that ILA promotes obesity by enhancing intestinal lipid absorption, promoting fatty acid uptake in white adipose tissue (WAT) and fat mass accumulation [40]. Because supplementation with dietary High BCAA had the most pronounced effect on tryptophan metabolism, we, therefore, decided to determine whether and how High BCAA feeding impacts metabolic health, particularly liver lipid metabolism. We next tested the hypothesis that High BCAA intervention, but not either the High Leu, High Ile or High LeuIle, significantly decreased hepatic lipid deposition, as evidenced by H&E and oil red O staining and accompanied by declined hepatic TG level (Fig. 3A–D). However, intake of either the High Leu, High Ile, High LeuIle or High BCAA diet did not affect liver weight and liver index (Figs. S4A and B), which can be attributed to the little change in food intake and body weight. To gain insight into the cellular morphological structure associated with lipid metabolism in response to a High BCAA diet, we utilized transmission electron microscopy (TEM) to detect differentially changed cellular ultrastructure. Intriguingly, the livers of hens fed Ctrl AA diet displayed partly damaged mitochondria and ER with ultrastructural abnormalities, abnormal mitochondrial morphology, and more LD, whose pathological features are similar to NAFLD in humans (Fig. 3A). This suggests enhanced hepatic steatosis and DNL, indicating that these hens can spontaneously develop NAFLD under a high-energy and high-protein dietary background. On the contrary, the High Leu diet intervention resulted in improved cellular mitochondria and ER structure, as well as organelle morphology (Fig. 3A). Interestingly, long-term exposure to a High Ile diet severely damaged cellular structures, including badly damaged mitochondria and ER with ultrastructural abnormalities, as well as deformed and spillover LD (Fig. 3A), further demonstrating the adverse and toxic metabolic effects of high dietary isoleucine [19]. High LeuIle feeding partially alleviated damaged mitochondria and ER with ultrastructural abnormalities compared to High Ile feeding (Fig. 3A). This is contrast with previous reports [9] that long-term High BCAA intervention significantly recovered damaged cellular structure, such as improved mitochondria and ER ultrastructure, and a dramatic reduction in the number of LD in the hepatocyte of hens (Fig. 3A–C). These data aligned with the reduction of hepatic TG (Fig. 3D). These results indicate that 67 % High BCAA feeding is non-toxic and ameliorates hepatic steatosis and hepatocellular injury.

**Fig. 4:**
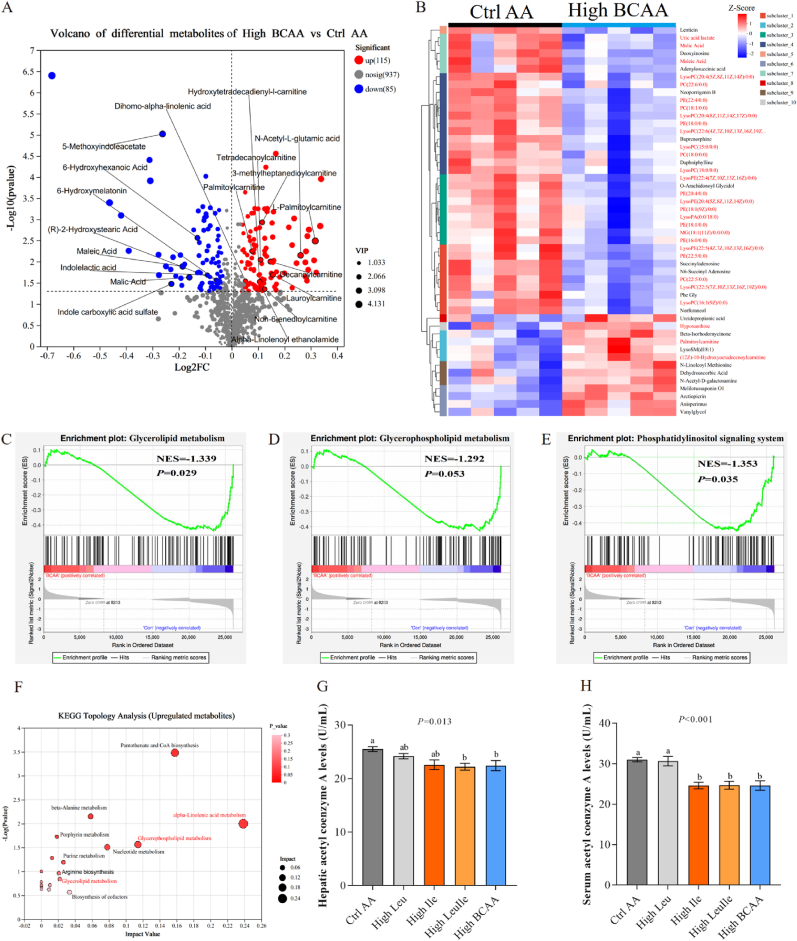
High BCAA diet reprograms hepatic lipidome (n = 5–6/group). (A) Volcano of differential metabolites between Ctrl AA and High BCAA groups. Differential metabolites of interest are visualized. (B) Heatmaps of top50 variable metabolites in the metabolomic analysis of Ctrl AA and High BCAA-fed hen livers. (C–E) GSEA enrichment analysis associated lipid metabolism signaling pathway. (F) KEGG enrichment analysis of upregulated metabolites from Ctrl AA and High BCAA-fed hen livers. (G) Hepatic acetyl coenzyme A. (H) Serum acetyl coenzyme A.

To gain insights into the changed lipid metabolism, we performed untargeted metabolomics to analyze the impacts of High BCAA intervention on liver metabolism. The metabolomic profile of High BCAA formed a distinct cluster away from that of Ctrl AA by PCA (PC1:25.90 %, 25.90 % and PC2: 15.80 %, 41.7 %) and PLS-DA analysis (Figs. S4C–E), and OPLS-DA loading plots showed that 1346 compounds were obtained from MS raw data (Fig. S4F). Among 1137 annotated metabolites, 10 % (115) were differentially upregulated between Ctrl AA and High BCAA livers (|fold change| ≥1, VIP_pre _OPLS-DA>1, *p*-value ≤0.05 by students t-test (Unpaired)), while 7 % (85) were differentially downregulated (Fig. 4A), respectively. It is noted that among all analyzed metabolites, High BCAA intervention induced the most changed metabolites in lipids, which is consistent with the key function of BCAA in regulating lipid metabolism in liver physiology [41]. The most profoundly altered metabolites in High BCAA feeding include elevated fatty acid catabolic products l-palmitoylcarnitine, lauroylcarnitine, 3-methylheptanedioylcarnitine, decanoylcarnitine, tetradecanoylcarnitine, non-6-enedioylcarnitine, palmitoylcarnitine, dihomo-alpha-linolenic acid, and alpha-linolenoyl ethanolamide, and reduced fatty acid synthesis substrates (R)-2-hydroxystearic acid, maleic acid, 6-hydroxyhexanoic acid, and malic acid (Figs. S4G–S). Looking at the top50 most dramatically changed metabolites between Ctrl AA and High BCAA groups, we identified that the High BCAA group appears to have >40 lipid species across several lipid subclasses such as diacylglycerols (DAGs), phosphatidylcholines (PCs), phosphatidylethanolamines (PEs), lysophosphatidylcholine (LysoPC), lysophosphatidylethanolamine (LysoPE), phosphatidic acids (PAs), Monoglycerides (MGs), lysophosphatidic acids (LysoPA), lysosphingomyelin (LysoSM), phosphatidylinositol (PIs), phosphoglycerides (PGs), and phosphatidylserine (PS) were decreased (Fig. 4B), which was evidenced by downregulated glycerolipid metabolism, phosphatidylinositol signaling system and glycerophospholipid metabolism (Fig. 4C–E). To examine these significant metabolites between lipid changes and host metabolism induced by a High BCAA diet, we enriched and analyzed these metabolites using KEGG and found enhanced pantothenate and CoA biosynthesis, phosphatidylinositol signaling system, alpha-linolenic acid metabolism, and glycerophospholipid metabolism, whereas declined citrate cycle (TCA cycle), ether lipid metabolism, and sphingolipid metabolism, respectively (Fig. 4F and Fig. S4T), suggesting decreased DNL and enhanced lipid catabolism. Consistent with reduced fatty acid synthesis substrates (R)-2-hydroxystearic acid, maleic acid, 6-hydroxyhexanoic acid, and malic acid, we found the reduction of acetyl-CoA in the liver and circulation of hens fed a High BCAA diet (Fig. 6G and H), a substrate of lipid synthesis, revealing inhibited DNL. Collectively, we demonstrate that a High BCAA diet alleviates hepatic steatosis by inhibiting DNL and reprograming hepatic lipidome.

**Fig. 5:**
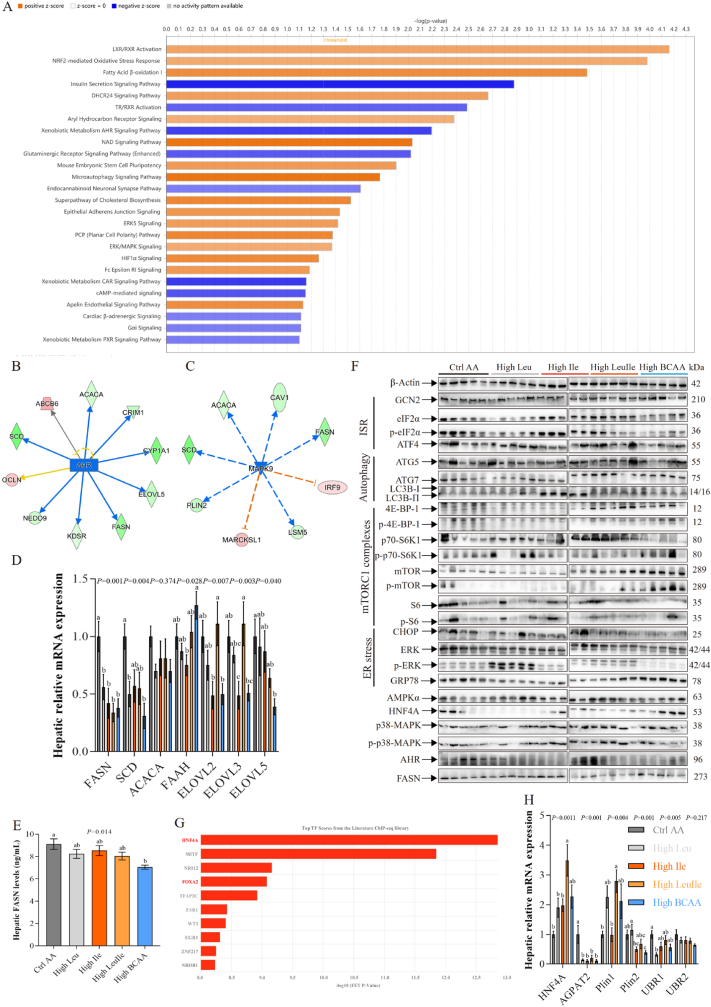
High BCAA diet inhibits AHR/MAPK9-mediated *de novo* lipogenesis and independent on GCN2 and mTORC1 (n = 5–6/group). (A) Enrichment comparison of metabolic and overall pathways based on RNA-seq data from Ctrl AA and High BCAA groups using the bioinformatics Ingenuity Pathway Analysis (IPA). Orange, positive z-score represent predicted activation. Blue, negative z-score represent predicted inhibition. Gray, no activity pattern available. (B–C) Upstream regulator molecule analysis. Orange line: leads to activation. Blue line: leads to inhibition. Yellow line: findings inconsistent with state of downstream molecule. Gray line: effect not predicted. Dashed lines: indirect relationship. Solid lines: direct relationship. Red frame: increased measurement. Green box: decreased measurement. (D) Hepatic mRNA expression involved in lipid synthesis. (E) Hepatic FASN protein levels. (F) Representative Western blot images (n = 5/group), ISR: Integrated stress response. (G) Significantly enriched transcription factors predicted to mediate the DEGs in the liver of fasted High BCAA-fed hens. (H) Hepatic mRNA expression of HNF4A target genes.

**Fig. 6:**
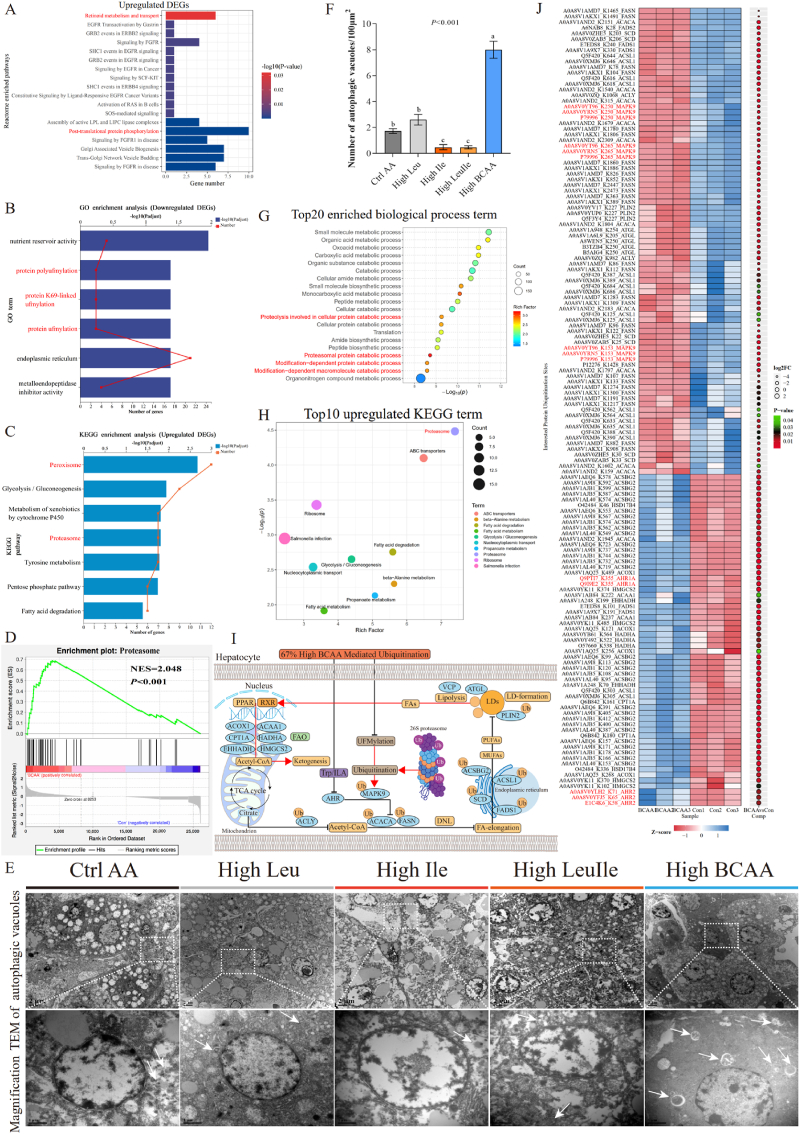
High BCAA diet activates MAPK9 ubiquitination by inhibiting UFMylation mediated ubiquitin-proteasome autophagy activation (n = 3–5/group). (A) Reactome enriched pathways of upregulated DEGs. (B) GO enriched pathways of downregulated DEGs. (C) KEGG analysis of upregulated DEGs. (D) GSEA analysis of proteasome. (E–F) The number of statistical autophagic vacuoles and representative images of hepatic TEM from High Leu, High Ile, High LeuIle or High BCAA feeding hens. (G) Top20 enriched biological process GO terms. (H) KEGG terms associated with proteins with increased ubiquitylation after High BCAA treatment. (I) Schematic illustrating High BCAA mediated protein ubiquitylation involved in lipid metabolism. Red line: leads to activation. Black line: leads to inhibition. (J) Hepatic heatmap of differential ubiquitylation sites from Ctrl AA or High BCAA-fed laying hens. Ubiquitylation sites of interest are visualized.

### 3.4 High BCAA diet inhibits AHR/MAPK9-mediated *de novo* lipogenesis

To better understand the mechanism of DNL downregulation through High BCAA, we next focused on our KEGG enrichment analysis and unveiled that drug metabolism-cytochrome P450 and the metabolism of xenobiotics by cytochrome P450 signaling pathways were significantly downregulated (Fig. 2F), suggesting that hepatic metabolism and elimination of xenobiotics, such as excessive nutrients, are essential for health. To get insight into potential signaling pathways in response to a High BCAA feeding, we used Ingenuity Pathway Analysis (IPA), an experimentally validated bioinformatics tool for signaling pathway enrichment analysis. We found the xenobiotic metabolism AHR signaling pathway and insulin secretion signaling pathway were significantly inhibited, suggesting that these pathways act as inhibitors of the observed DEGs changes (Fig. 5A). As insulin target organs, such as adipose tissue, skeletal muscle and the liver, are directly affected by BCAA., Intake of high BCAA exacerbates the development of IR, and with increased blood levels of BCAA correlated with IR [7]. For instance, pancreatic β cells can sense circulating leucine to stimulate insulin secretion, which is important for glucose metabolism [42]. Therefore, we first tested whether High BCAA feeding impacts host glucose and insulin metabolism. We found that the glucose transporter (GLUT4) was downregulated by the High BCAA diet, but did not affect BCAA-associated transporters and the levels of glycogen content in the liver and muscle, and total amino acid, insulin, and glucagon in the pancreas (Figs. S5A–C). Intriguingly, glucose concentration in the pancreas was elevated by High BCAA feeding and accompanied by a decline in hepatic glucagon levels, but it did not impact fasted serum, hepatic and pancreatic insulin and glucose levels (Fig. S5D-L). Consistently, we also observed significantly decreased pancreatic trypsin enzyme activity (Figs. S5M and N). FGF19/21, as a hormone, enhances hepatic insulin sensitivity and maintains energy homeostasis, is induced by a variety of stresses [[43], [44], [45], [46], [47]]. We found that a High BCAA diet did not impact fasted serum, ileum and hepatic FGF19 levels (Fig. S5O). However, we observed downregulated FGF1 and FGF23 after High BCAA intervention (Fig. 1F), suggesting that the metabolic impacts of a High BCAA diet are independent of FGF19, and the role of FGF1/FGF23 remains unclear. The PI3K-Akt pathway also participates in regulating glucose and lipid metabolism. For instance, glucose uptake mediated by isoleucine depends on PI3K but not mTOR and decreases plasma glucose levels [48]. The PI3K-Akt signaling pathway, as evidenced by GSEA enrichment analysis (Fig. S5P), which reflects glucose utilization, was downregulated in hens consuming a High BCAA diet, suggesting inhibited glucose utilization. However, we observed enhanced glycolysis or gluconeogenesis in the liver of hens fed a High BCAA diet (Fig. S1J), which is validated by upregulated lactate dehydrogenases LDHA and LDHB expression (Fig. 1F), suggesting upregulated glucose metabolism. Collectively, these results suggest that High BCAA improves hepatic insulin sensitivity by activating glycolysis or gluconeogenesis to maintain insulin homeostasis.

The hepatic xenobiotic metabolism machinery regulates the biotransformation and elimination of various exogenous substances such as overnutrition, thus guaranteeing homeostasis and health [49]. To determine which genes and/or proteins were responsible for DNL, we further used upstream regulated factor analysis and found two inhibited regulators, including aryl hydrocarbon receptor (AHR) and mitogen-activated protein kinase 9 (MAPK9) (Fig. 5B and C), which are involved in lipid metabolism. Lipid metabolism related genes were directly regulated by AHR, while were indirectly regulated by MAPK9 (Fig. 5B and C), suggesting the inhibition of AHR/MAPK9 signaling pathway mainly affects lipid metabolism. Mice with liver-specific knock-out AHR exhibited enhanced DNL activity, severe hepatic steatosis, inflammation and injury [50], while activating hepatic AHR by 5-HIAA can alleviate the pathogenesis of T2D [51]. The MAPK signaling pathway is closely involved in the onset and development of obesity and IR [52], and inhibition of MAPKs is related to less inflammatory cell infiltration, improved glucose tolerance, and ameliorated adipocyte cell diameter [53]. The AHR/MAPK9 signaling pathway mainly participates in regulating lipid metabolism-associated genes, including fatty acid synthesis genes such as FASN, SCD, ACAA and CYP1A1, as well as fatty acid elongation gene ELOVL5, and LD formation gene PLIN2, indicating that these genes are prioritized and regulated, thereby influencing hepatic lipid synthesis (Fig. 5B and C). RNA-Seq DEGs further confirmed that these genes involved in lipogenesis were significantly downregulated by High BCAA feeding compared with Ctrl AA (Fig. 1F) and qPCR validation revealed markedly downregulated AHR and MAPK9 target gene mRNA expression involved in the fatty acid synthesis and elongation (Fig. 5D). ELISA analysis also uncovered the reduction of hepatic FASN protein secretion (Fig. 5E). Accordingly, as shown in Fig. 5F, western blotting further confirmed reduced hepatic FASN protein expression (Fig. S6H), which is line with downregulated FASN gene expression and decreased protein secretion. These data reinforced the critical role of AHR and MAPK9 for DNL in the liver. Additionally, we found that hens fed either the High Leu, High Ile, High LeuIle or High BCAA had no effect on host cholesterol and bile acid metabolism-related indexes and other lipid synthesis-associated enzymes in the liver and circulation (data not shown). Together, these results demonstrate that inhibited DNL was mediated by the inhibition of AHR and MAPK9.

Given that BCAA are widely known as mTORC1 agonists [[54], [55], [56], [57], [58]], while the integrated stress response (ISR) associated GCN2/ATF4 and its downstream effector FGF21 sense amino acid deficiency [19,59,60]. GCN2, one of the major amino acid-sensing kinases, which can be activated by uncharged tRNAs, is necessary for the adaptation to amino acid deprivation [[61], [62], [63]]. We identified decreased phosphorylation of the GCN2 substrate eIF2α in hens fed a High BCAA diet, but surprisingly no impact on the expression of its downstream transcription factor ATF4 (Fig. 5F and Fig. S6A). As mTORC1 agonists, we were surprised that only High BCAA feeding altered the protein expression of mTOR, S6, as well as the phosphorylation of mTOR, but did not influence the phosphorylation of S6 that is a downstream readout of mTORC1, or the mTORC1 substrates 4E-BP-1 and S6K1 (S6 kinase 1), in the liver (Fig. 5F and Figs. S6B–E). The activity of hepatic GCN2 and mTORC1 is also not necessary for the metabolic impacts of a low isoleucine diet [19]. Given both the Western blot data and the previous reports, we demonstrate that High BCAA intervention does not ameliorate hepatic steatosis via activating hepatic GCN2 or mTORC1. The activation of AHR promotes obesity and IR [64], whereas impaired AHR leads to dietary- and genetic-mediated metabolic impairments, notably glucose dysmetabolism and liver steatosis [65,66]. As shown in Fig. 5D, inhibition of AHR/MAPK9-mediated lipid synthesis related genes downregulation to promote metabolic health, we next determined the protein levels of AHR and p38-MAPK. We found that High BCAA feeding significantly declined hepatic AHR protein expression but only partly reduced p38-MAPK protein expression (Fig. 5F and Figs. S6E–H). We conclude that the metabolic impacts of a High BCAA intervention are directly dependent on AHR and that some, but not all, are indirectly mediated by MAPK9. Consistently, our RNA-seq and hepatic metabolomic also indicated downregulated tryptophan metabolism (Fig. 2F and J), which is associated with the reduced tryptophan absorption mediated by High BCAA, restraining AHR ligand ILA production that can activate the AHR.

To get insight into latent transcriptional regulators in response to a High BCAA intervention, we conducted a recently developed and experimentally validated tool ChEA3 that is used for transcription factor enrichment analysis [67]. We found the top10 significantly enriched transcription factors predicted as drivers of the observed DEGs (Fig. 5G). Intriguingly, among these transcription factors, HNF4A and FOXA2 were previously proved as regulators of hepatic lipid metabolism and responses to a low isoleucine diet [19,68]. It is interesting that HNF4A is the most enriched transcription factor and was activated by High BCAA feeding, which target genes that are downregulated the most by a High BCAA diet (Fig. 1F). Among the top20 downregulated genes are lipid metabolism-related AGPAT2, CYP1A1 and FASN. AGPAT2 is a key gene that catalyzes the acylation of lysophosphatidic acid to generate phosphatidic acid (PA) and is required for the synthesis of phospholipids and TG [69]. qPCR and Western blot data evidenced increased HNF4A mRNA and protein expression levels and downregulated LD formation critical genes PLIN2 and AGPAT2 expression (Fig. 5F and H and Fig. S6F), which is consistent with decreased hepatic TG, demonstrating that High BCAA feeding activated the transcription factor HNF4A that inhibited the formation of LD. Yet, we found High BCAA feeding had little impact on the mRNA expression of UBR1/2 (Fig. 5H), which has been confirmed can be powerfully bound and activated by dietary leucine and isoleucine [27]. We revealed that some, but not all, of the metabolic impacts of High BCAA intervention are dependent on the transcription factor HNF4A mediated LD formation. In summary, these results jointly demonstrate that the inhibition of AHR/MAPK9 mediated by tryptophan-ILA ligand depletion is both sufficient to ameliorate NAFLD-related hepatic steatosis and required for a High BCAA to maximally ameliorate metabolic health.

### 3.5 High BCAA diet activates MAPK9 ubiquitination by inhibiting UFMylation mediated ubiquitin-proteasome autophagy activation

As shown in Fig. 5, we have shown that amino acid-sensitive GCN2 and rapamycin-insensitive mTOR signaling is not critical for High BCAA-induced DNL inhibition. As illustrated in Fig. 5, AHR was directly inhibited by High BCAA feeding, while MAPK9 was indirectly inhibited, demonstrating the inhibition of AHR was directly mediated by tryptophan-ILA depletion, and the inhibition of MAPK9 is dependent on other potential mechanisms. To better understand the underlying mechanism of High BCAA inhibits DNL through MAPK9, we combined gene-gene interaction networks with signaling pathway over-representation examines on the 1058 DEGs. Reactome enriched pathways based on the Reactome database unveiled that the enriched signaling pathways were referred not only to fatty acid metabolism but also to post-translational protein phosphorylation pathway (Fig. 6A), suggesting that High BCAA intervention might activate post-translational regulatory processes. Interestingly, our IPA analysis identified the activation of micro-autophagy signaling pathway (Fig. 5A), which plays a critical role in lipid mediated autophagy (lipophagy) [70]. Ubiquitination, a type of micro-autophagy, is one of the most familiar post-translational modifications in eukaryotes and plays ubiquitous roles in lipid metabolism [71]. Interestingly, we observed downregulated biological processes associated with protein polyufmylation, protein K69-linked ufmylation and protein ufmylation based on the GO analysis of downregulated DEGs (Fig. 6B), unveiling the downregulation of UFMylation. As with ubiquitination, the ubiquitin-fold modifier 1 (UFM1) conjugation system is one of ubiquitin-like modification that UFMylation is catalyzed by the E1-like ubiquitin-like modifier activating enzyme 5 (UBA5) and E2-like ubiquitin-fold modifier-conjugating enzyme 1 (UFC1), and by the E3-like ligase UFM1-specific ligase 1 (UFL1) [[72], [73], [74]]. Consistently, we found that High BCAA feeding significantly downregulated these genes (Fig. 1F), demonstrating the inactivation of UFMylation. As an important post-translational modification, UFMylation is necessary for the maintenance of essential biological functions via antagonizing its ubiquitination and promoting proteasome degradation [75]. Inhibition of UFMylation induced by High BCAA intervention suggests the activation of ubiquitination. KEGG and GSEA analysis all revealed the activation of the proteasome (Fig. 6C and D) and TEM further confirmed the enhanced formation of autophagic vacuoles and increased numbers (Fig. 6E and F), indicating the activation of ubiquitin-proteasome autophagy. To determine how High BCAA impacts protein function, we also investigated protein function based on MS data using the OECloud tools (https://cloud.oebiotech.com). In both Ctrl AA and High BCAA feeding hens, we identified clusters containing proteins related to the KEGG oxidative phosphorylation, valine, leucine and isoleucine degradation, PPAR signaling pathway, glycolysis or gluconeogenesis, as well as translational changes in the proteasome, peroxisome, ribosome, and spliceosome (Figs. S7A–D). This data suggests that inhibition of UFMylation induced by High BCAA feeding is required for the activation of ubiquitin-proteasome autophagy but the potential mechanism and its underlying biological functions in lipid metabolism remain poorly understood.

To further elucidate the important role of ubiquitylation in controlling lipid metabolism after High BCAA intervention, we conducted ubiquitylation site profiling analysis using data independent acquisition (DIA) quantitative ubiquitin-modified proteomics. In three independent samples, PCA analysis unveiled a distinct cluster away between Ctrl AA and High BCAA groups (Fig. S7E) and Pearson's correlation coefficients and Log2 scaled intensity revealed high similarity among bio-replicated samples (Figs. S7F and G). We identified 4650 protein groups, 22,914 unique peptides, and 13,794 ubiquitin sites among these six samples, respectively (Fig. S7H). These proteins are mainly distributed in the cytoplasm (46.08 %) and membrane (21.62 %) (Fig. S7I). GO analysis based on identified proteins revealed the enrichment of metabolic processes related to small molecule, peptide metabolic process, organic acid, oxoacid, organic substance catabolic process, carboxylic acid, cellular amide, monocarboxylic acid, catabolic process and small molecule biosynthetic process (Fig. S8A). We also observed significantly enriched proteolysis involved in cellular protein catabolic process, modification-dependent protein catabolic process, proteasomal protein catabolic process, and modification-dependent macromolecule catabolic process among top20 GO biological process (Fig. 6G). The proteasomal protein catabolic process is the most enriched (Fig. 6G), unveiling High BCAA intervention mainly activates proteasome-mediated protein degradation. KEGG functional annotation analysis furthermore proved our RNA-Seq results that concomitant with a substantial overlap enrichment across peroxisome, proteasome, tryptophan metabolism, glycolysis or gluconeogenesis, metabolism of xenobiotics by cytochrome P450, fatty acid metabolism and degradation, and PPAR signaling pathway (Fig. S8B). Notably, among the top 20 most enriched KEGG terms, we also found the enrichment of protein processing in the endoplasmic reticulum, ubiquitin-mediated proteolysis and the proteasome was still the most enriched (Fig. S8C), uncovering the activation of ubiquitin-proteasome autophagy axis.

We quantified the protein abundance of more than 13,119 reliable ubiquitination sites among three biological replicate samples. High BCAA intervention produced profound changes in the ubiquitin-modified proteome log2 fold change ratio (2-fold increase): 1581 ubiquitylation sites on 472 proteins displayed significant increase in ubiquitylation, and 1311 sites on 307 proteins displayed a decrease, as evidenced by volcano overlap (Fig. S8D). We found most enriched ubiquitination sites in lipid metabolism-associated proteins, including fatty acid synthesis, elongation, oxidation, lipolysis, lipoprotein efflux and LD formation between High BCAA and Ctrl AA response. GO analysis of significant sites related proteins revealed the enrichment of post-Golgi vesicle-mediated transport, ribosomal large subunit assembly, clathrin-coated vesicle membrane, peroxisomal membrane, microbody membrane, clathrin-coated endocytic vesicle, cytosolic large ribosomal subunit, centriole, coated vesicle, clathrin-coated vesicle, clathrin adaptor activity, and cargo adaptor activity (Figs. S8E–G), unveiling activation of clathrin-mediated endocytosis which contributes to lysosomal transportation mediated lysosome-targeting degradation of extracellular proteins [76]. Furthermore, as for KEGG functional categorization, fatty acid degradation and metabolism, proteasome, and glycolysis or gluconeogenesis were upregulated and the proteasome was the most enriched pathway among top10, whereas fatty acid biosynthesis was the most downregulated (Fig. 6H and Figs. S9H and I), pointing to the direct involvement of ubiquitin-proteasome mediated autophagy degradation. We classified altered sites according to their role in lipid metabolism which were (1) fatty acid synthesis, (2) elongation, (3) oxidation, (4) lipolysis, (5) lipoprotein efflux, and (6) LD formation. As for functional categorization, fatty acid synthesis (FASN, ACACA, SCD, and ACLY), elongation (ACSL1, ACSBG2 and FADS1) and LD formation (PLIN2) related proteins were widely ubiquitination, while fatty acid oxidation (HSD17B4, CPT2, CPT1A, ACOX1, ACAA1, EHHADH, HADHA, HMGCS2, ALDH1A1, and ALDH7A1) and lipolysis and lipoprotein efflux (ATGL, VCP and APOB) related proteins were significantly deubiquitination by High BCAA intervention (Fig. 6I). Intriguingly, among these sites, we found that High BCAA induced a deubiquitination degradation increase of AHR-linked sites such as AHR1A_K355, AHR2_K58, AHR2_K71 and AHR2_K65, whereas MAPK9-linked sites including MAPK9_K265, MAPK9_K250, and MAPK9_K153 were all ubiquitinated by High BCAA feeding (Fig. 6J), indicating the inhibition of AHR and MAPK9 pathways are mutually independent that AHR was directly inhibited by tryptophan-ILA depletion but the MAPK9 was dependent on ubiquitination degradation. Furthermore, we found that FASN, ACACA, SCD, and ACLY, which are directly related to DNL, were widely ubiquitinated by High BCAA intervention contribution to the restrained lipid synthesis (Fig. 6J), pointing to the direct participation in the ubiquitin-proteasome autophagy in lipid synthesis related protein degradation. Among these lipid synthesis related proteins, we noted that FASN sites including FASN_K1465, FASN_K1491, FASN_K1860, FASN_K1886, FASN_K826, FASN_K852, FASN_K2447, FASN_K2473, FASN_K78, FASN_K104, FASN_K1780, FASN_K1806, FASN_K86, FASN_K112, FASN_K107, FASN_K133, FASN_K1274, FASN_K1300, FASN_K1191, FASN_K1217, FASN_K96 and FASN_K122 were extensively ubiquitinated by High BCAA feeding, which is concomitant with our RNA-Seq, Western blot and ELISA data, demonstrating FASN ubiquitination degradation induced by High BCAA feeding is necessary for the amelioration of NAFLD. Thus, these data consistently highlighted the potential role of MAPK9 in inhibiting DNL was triggered by the activation of ubiquitin-proteasome autophagy mediated K265, K250 and K153 sites ubiquitination. Together, our detailed analysis unveiled a wide protein degradation of High BCAA feeding-induced ubiquitylation, including DNL, fatty acid elongation and LD formation-related proteins, and activating lipolysis and fatty acid oxidation-related proteins expression and potential new regulators.

### 3.6 High BCAA diet promotes hepatic lipid catabolism by activating PPAR-RXR

**Fig. 7:**
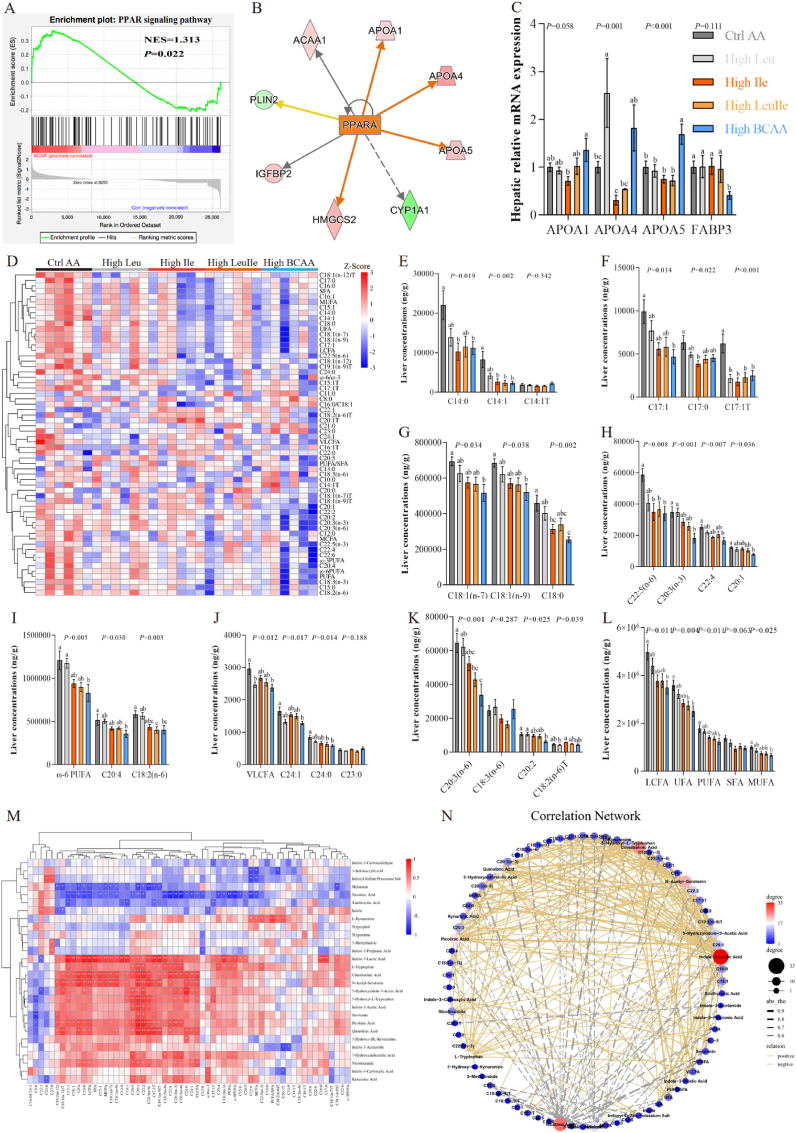
High BCAA diet promotes hepatic lipid catabolism by activating PPAR-RXR (n = 5–6/group). (A) GSEA enrichment analysis of PPAR signaling pathway. (B) PPAR targets regulated molecules. Orange line: leads to activation. Yellow line: findings inconsistent with state of downstream molecule. Gray line: effect not predicted. (C) Hepatic mRNA expression of PPAR target genes involved in lipid synthesis and transportation. (D) Heatmaps of hepatic fatty acid profile. (E–L) Hepatic fatty acid levels. (M − N) Serum tryptophan metabolites and hepatic fatty acid Spearman's correlation analysis.

We further to determine which pathways were responsible for lipid catabolism because increased fatty acid catabolic products fatty acyl-carnitines. Among all metabolic pathways analyzed, in particular, alpha-linolenic acid metabolism is activated by High BCAA intervention (Fig. 4F), suggestive of enhanced fatty acid catabolism or β-oxidation (Fig. 5A), perhaps in response to increased fatty acyl-carnitines. To investigate potential signaling pathway-induced changes in fatty acid metabolism, we performed KEGG and GSEA enrichment analysis and identified the activation of the PPAR signaling pathway (Fig. 7A and Fig. S1J), likely reflected by increased ketogenesis and fatty acyl-carnitines. It is consistent with RNA-Seq analysis that our protein function analysis based on MS data and ubiquitin-modified proteomics all confirmed the activation of the PPAR signaling pathway (Fig. S7 and Fig. S8). qPCR validation confirmed the significantly upregulated mRNA expression of PPAR target genes (Fig. 7B and C), which promote lipolytic fatty acid output into circulation. PPAR is activated by free fatty acids and then undergoes a conformational change that enhances their heterodimerization with retinoid X receptors (RXRs) [71,77]. The PPAR-RXR complex assembles at PPAR response elements (PPREs) and accelerates the transactivation of target genes [71]. We also observed High 10.13039/100022047BCAA feeding activated retinol metabolism and fatty acid degradation by the activation of LXR/RXR (Fig. 5, Fig. 6A), resulting in inhibited 10.13039/501100016091DNL and enhanced fatty acid β-oxidation in mitochondria, which was supported by 10.13039/501100001838TEM that unveiled completed mitochondrial morphology and increased 10.13039/100031136LD unsaturated degree (Fig. S9A). To determine potential long-term intake of High BCAA diet-mediated changes in fatty acids, we conducted targeted metabolomics and identified significant fatty acid profile changes between the livers of hens fed either the High Leu, High Ile, High LeuIle or High BCAA (Fig. 7D). Consistent with RNA-Seq and untargeted metabolomics analysis, we found the greatest abundance of changes in hepatic long-chain fatty acids (LCFA) and very long-chain fatty acids (VLCFA) (Fig. 7E-L), and the fewest in ω-3 polyunsaturated fatty acids (PUFA), with little changes in medium-chain fatty acids (MCFA) between the High Leu, High Ile, High LeuIle or High BCAA-fed hens (Fig. S9B-M). Among changed fatty acids, hepatic LCFA including C14:0, C14:1, C15:1, C17:1, C17:1T, C18:0, C19:1 (n-9)T, C18:1 (n-7), C20:1, C18:1 (n-9), C20:2, C18:1 (n-12), C20:3 (n-3), C18:1 (n-12)T, C20:3 (n-6), C22:4, C18:2 (n-6), C20:4, C22:2 and C22:5 (n-6), VLCFA concluding C24:0 and C24:1, and total ω-6PUFA, LCFA, VLCFA, unsaturated fatty acids (UFA), PUFA and monounsaturated fatty acids (MUFA) were dramatically reduced by High BCAA intervention, this is in alignment with enhanced fatty acid β-oxidation and increased fatty acyl-carnitines. In summary, we found that consumption of a High BCAA diet specifically activates hepatic lipid catabolism by activating PPAR-RXR mediated fatty acid β-oxidation. In contrast to our original hypothesis that a High BCAA diet would bring adverse impacts on NAFLD, we indicate that the metabolic impacts of a High BCAA diet were largely beneficial. Furthermore, isocaloric and iso-proteinic dietary macronutrient background may be an extremely key factor in the metabolic response to a High BCAA diet.

In addition, we also found trends to increased energy expenditure by activating oxidative phosphorylation and pentose phosphate pathway (Figs. S9N and O), as evidenced by increased hepatic acyl-carnitines and elevated hepatic and circulating ATP (Figs. S9P and Q). Similarly, we also found High BCAA intervention activated glutathione metabolism and NAD signaling pathway (Fig. 5A and Fig. S1J), whereas hepatic NAD^+^ level was decreased and accompanied by increased NADH/NAD^+^ ratio (Figs. S9R and S), revealing increased energy expenditure. Given the decreased tryptophan metabolites of AHR ligand ILA in High BCAA-fed hens, we examined the possibility that these declined hepatic fatty acids were mediated by circulating amino acid which further led to the tryptophan metabolites reduction, by using Spearman's correlation. We correlated serum amino acid, tryptophan metabolites and hepatic fatty acids from Ctrl AA and High BCAA and plotted these in a clustered heatmap. We identified High BCAA had a great impact on UFA, PUFA, MUFA, saturated fatty acids (SFA), ω-6PUFA and LCFA that PUFA/SFA was negatively correlated (Figs. S10A and B), uncovering increased lipid unsaturated degree. These decreased hepatic fatty acids were positively associated with decreased circulating tryptophan and total AAA (Figs. S10C and D). These declined tryptophan metabolites such as ILA, cinnabarinic acid, N-acetyl-serotonin, picolinic acid, quinolinic acid and 5-HIAA, in particular, having positively stronger correlations of declined hepatic fatty acids (Fig. 7M and N). Notably, ILA was the most positively correlated with the loss of LCFA, MUFA, UFA and SFA, suggestive of the reduction of hepatic fatty acid was contributed to the inhibition of AHR induced by High BCAA-mediated AHR ligand ILA depletion. Overall, in combination with the results of Fig. 5, Fig. 6, these data strongly demonstrate that High BCAA feeding is both sufficient to inhibit hepatic lipid synthesis via inhibition of AHR/MAPK9 mediated DNL and activating PPAR-RXR mediated fatty acid β-oxidation, which is necessary for a High BCAA diet to maximally ameliorate hepatic steatosis.

### 3.7 High BCAA diet activates pexophagy to promote fatty acid β-oxidation

**Fig. 8:**
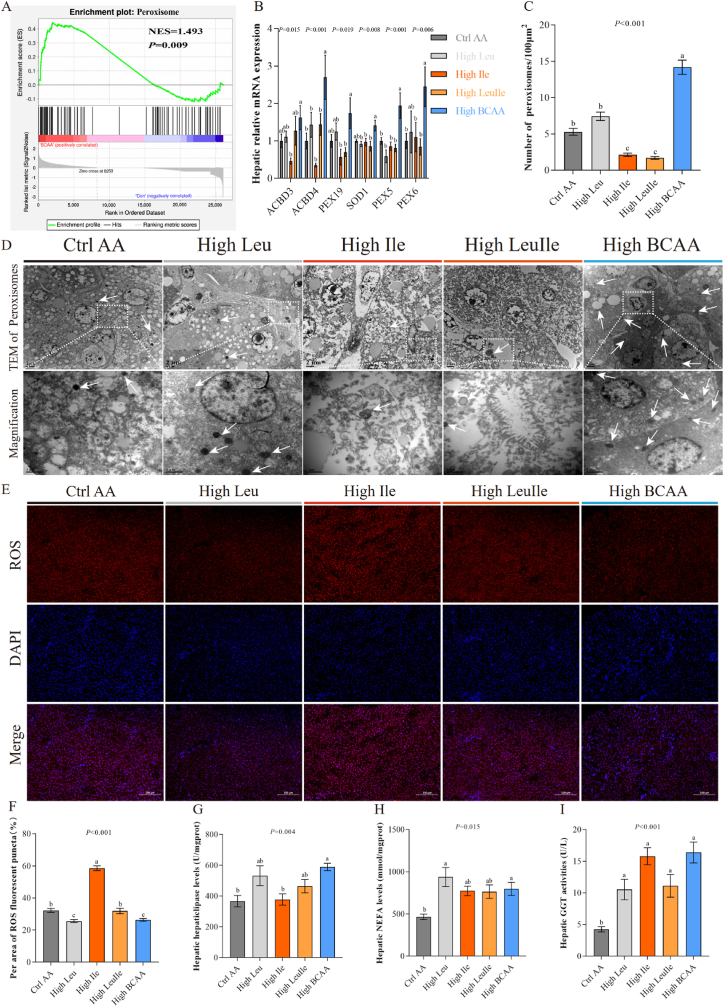
High BCAA diet promotes fatty acid β-oxidation via activating pexophagy (n = 5–6/group). (A) GSEA analysis of peroxisome. (B) Hepatic mRNA expression involved in peroxisomal biogenesis and fatty acid metabolism. (C–D) Representative images of hepatic TEM and the number of statistical peroxisomes from High Leu, High Ile, High LeuIle or High BCAA-fed laying hens. (E–F) Representative images of hepatic ROS immunofluorescence and statistical area from High Leu, High Ile, High LeuIle or High BCAA feeding hens. (G–I) Hepatic lipase, NEFA and GGT.

As illustrated in Fig. 6, KEGG analysis of upregulated DEGs found the most enriched pathway was peroxisome, which was also confirmed by GSEA enrichment analysis (Fig. 8A), revealing peroxisomes also participate in lipid metabolism but not just an organelle. qPCR validation from the livers evidenced High BCAA feeding significantly upregulated the gene expression levels of peroxisomal biogenesis factors and fatty acid catabolism, including ACBD3/4, PEX5/6 and PEX19 (LOC107049500) (Fig. 8B) [78]. Using TEM, we found High BCAA intervention significantly increased the number of peroxisomes (Fig. 8C and D), which is consistent with our RNA-Seq. Peroxisomes are dynamic organelles with essential roles in regulating all kinds of important metabolic activities such as the synthesis and turnover of complex lipids and are fully responsible for VLCFA β-oxidation, branched-chain fatty acids (BCFA) α-oxidation, and ether lipids synthesis [79]. Similarly, we also observed a reduction of VLCFA in the liver of High BCAA-fed hens and declined peroxisomal lipid metabolites such as PUFA (Fig. 7 and Fig. S9). In addition, peroxisomes are also required for ROS detoxification and amino acid metabolism [80,81]. Our immunofluorescence staining demonstrated High BCAA intervention significantly reduced hepatocyte ROS era compared to Ctrl AA (Fig. 8E and F). Peroxisomes also take part in lipolysis during activation of lipolysis by recruiting and regulating the main lipid droplet lipase ATGL [82,83]. Consistently, we found that High BCAA feeding activated hepatic lipase and its catabolic intermediates non-esterified fatty acid (NEFA) in the liver and circulation and accompanied by elevated gamma-glutamyltransferase (GGT) (Fig. 8G–I and Figs. S10E and F), demonstrating enhanced lipolysis. It is further validated by ubiquitin-modified proteomics that lipolysis and lipoprotein efflux related proteins including ATGL, VCP and APOB were significantly activation by High BCAA feeding (Fig. 6I), which can be partially explained by enhanced lipolysis via stimulating pexophagy. Recent research reported that hepatic peroxisomal β-oxidation derived acetyl-CoA restrains autophagy and accelerates steatosis by activating mTORC1 [84]. However, we did not observe the activation of mTORC1, evidenced by Western blot (Fig. 5F). High 10.13039/100022047BCAA intervention also had no effect on classical macro-autophagy validated by Western blot (Fig. 5F), and micro-mitophagy which was supported by downregulated PINK1-PRKN mediated mitophagy, mitophagy and receptor mediated mitophagy (data not shown). Taken together, these results suggest that High BCAA feeding stimulates hepatic fatty acid β-oxidation is partially mediated by pexophagy.

Given the downregulated tryptophan metabolism and the reduction of AHR ligand ILA production, we next examined the possibility that inhibition of AHR/MAPK9 impacts inflammatory response, oxidative stress, ER stress, and autophagy, which are important events during NAFLD [2]. High BCAA intervention did not affect the expression of autophagy-associated marker proteins such as ATG5, ATG7, LC3B–I, LC3B-II and LC3-II/LC3-I ratio (Fig. 5F and Fig. S6). This is because inhibition of autophagy by knockout of ATG7 or knock-down of ATG7 or ATG5 promotes TG storage in LD [85], suggesting that the beneficial metabolic impacts of High BCAA are independent of autophagy. In addition, lipids excess also inhibits AMPK- and mTOR-mediated autophagy by directly phosphorylating ULK1, leading to increased hepatic oxidative stress [86]. However, High BCAA intervention did not affect AMPKα expression and p-mTOR/mTOR ratio, although it activated the protein expression of p-mTOR and mTOR, suggesting AMPK-mTOR pathway is not involved in NAFLD-associated autophagy (Fig. 5F and Fig. S6). GCN2 promotes myocardial oxidative stress under stress conditions via activating ATF4, whereas GCN2 deficiency protects mice from HFD-induced oxidative stress, TG accumulation, and ER stress [87,88]. Consistently, as shown in Fig. 5F, High BCAA intervention significantly downregulated the expression of EIF2AK2 (PKR) gene and the protein levels of ER stress markers including GRP78, eIF2α, p-eIF2α, ERK, p-ERK, as well as the ratios of p-eIF2α/eIF2α and p-ERK/ERK, whereas it did not impact C/EBP homologous protein (CHOP) level [89] (Fig. 1F and Fig. S6), uncovering that High BCAA intervention ameliorated NAFLD-related ER stress. We further examined the possibility of liver inflammatory response under lipid loading and DNL, as AHR ligand ILA could enhance immune responses [90], using tissue staining. Sirius red and Masson staining found High BCAA reduced hepatic fibrogenesis and hydroxyproline levels by 41 and 56 % (Figs. S10G–I), indicating amelioration of liver injury and fibrosis. Furthermore, we found High BCAA intervention elevated the biomarker of liver cell injury [91] including hepatic alanine aminotransferase (ALT), serum aspartate aminotransferase (AST), AST/ALT ratio, and pro-inflammatory cytokines IL-1β and IL-6 (Figs. S11A–H), and accompanied by increased serum and hepatic GGT (Fig. 8G–I and Figs. S11E and F), which can be explained by enhanced hepatic lipolysis induced hepatic and circulating NEFA elevation. Increased reactive oxygen species (ROS) and impaired antioxidant defense system (including GSH, GSH-Px, SOD, and CAT, which mediated free radical scavenging and maintaining the intracellular redox balance) have been suggested to be highly associated with NAFLD pathogenesis [92,93]. During NAFLD, High BCAA intervention significantly activated antioxidant oxidases GSH-Px, T-AOC, and T-GSH in liver and serum GSH, T-GSH, GSH/GSSG ratio and total amino acid (Figs. S11I–AB), while accompanied by elevated hepatic MDA (Fig. S11M), which can be explained by enhanced hepatic lipolysis. Collectively, an 8-week High BCAA intervention partly improved liver injury, ER stress, oxidative stress and related metabolic disorders in laying hens with NAFLD, although accompanied by elevated NEFA. Together, these results suggested that High BCAA diet ameliorate NAFLD and associated metabolic disorders via activating pexophagy mediated fatty acid β-oxidation.

### 3.8 High BCAA diet fuels lipogenesis in adipose tissues

The well-known function of BCAA in hepatic lipid metabolism has been demonstrated but the potential role of High BCAA in adipose tissue of NAFLD-associated hens remains vague. Adipose tissue is important for lipid homeostasis by storing excess nutrients in LD and releasing bioenergetic substrates via lipolysis. For example, BCAA and their catabolites BCKAs regulate WAT browning and fuel adipocyte differentiation and lipogenesis [[94], [95], [96]]. However, we found either fed the High Leu, High Ile, High LeuIle or High BCAA diet had no impact on abdominal fat weight and abdominal fat index (Figs. S12A–C). Yet, we observed High BCAA feeding increased average adipocyte diameter (Fig. S12D), which was not mediated by enhanced BCAA uptake and catabolism because High BCAA intervention did not affect BCAA transporter expression and downregulated BCAA catabolic key gene BCAT1 compared to Ctrl AA (Fig. S12E). We also did not find changed gene mRNA expression such as fatty acid transportation and LD formation after High BCAA feeding (Fig. S12F). Interestingly, High BCAA intervention triggered the elevation of lipogenesis enzymes such as FASN, but not including ACC and ACLY, and accompanied by declined lipolysis enzyme ATGL (Figs. S12G and H). Consistently, High BCAA feeding decreased lipoprotein lipase (LPL) and increased VLDL and TG levels (Figs. S12I–K), but did not impact abdominal NEFA and lipogenesis substrates acetyl-CoA (Figs. S12L and M), suggesting enhanced abdominal lipid deposition that triggers adipocyte hypertrophy. We speculate that the increased adipocyte diameter is not mediated by increased abdominal fat BCAA uptake and catabolism; instead, it can be explained by FASN derived lipid synthesis mediated by monomethyl branched-chain fatty acids (mmBCFAs) that are *de novo* synthesized by mitochondrial BCAA catabolism, and then export to the cytosol by carnitine acetyltransferase that is specifically expressed in adipose, and finally elongated by FASN [96]. Brown adipose tissue (BAT) can transport BCAA into mitochondria via SLC25A44 and then effectively utilize BCAA for thermogenesis and promote systemic BCAA catabolism under cold exposure [97]. The impacts of High BCAA feeding on the metabolism of BCAA in adipose tissue of hens with NAFLD and their interaction remain further studied. Although the abdominal fat weight and abdominal fat index are not different, our results demonstrate that DNL via FASN is the primary driver of adipocyte hypertrophy.

## 4 Discussion

**Fig. 9:**
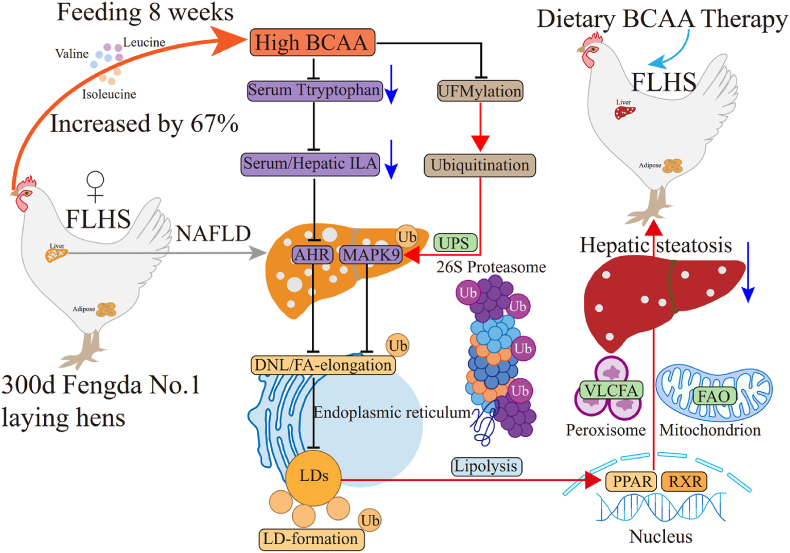
Schematic overview of the putative mechanism by which 67 % high BCAA affect the *de novo* lipogenesis and fatty acid β-oxidation of NAFLD in laying hens. Red line: leads to activation. Black line: leads to inhibition. Blue line: leads to a decrease.

Recently, it has become clear that dietary BCAA restriction improves metabolic health in rodents and humans [18,19], intaking of excessive BCAA accelerates hyperphagia and obesity and shortens lifespan [9], conversely. Recent reports indicated that a restriction of 67 % three BCAA is enough to recapitulate the advantage of a low protein diet, and dietary isoleucine restriction is the most efficient of these in the modulation of metabolic health [19]. However, three BCAA are often combusted together and with similar catabolic pathways, thus they are often studied as a group, rather than individually. We have shown that long-term intake of dietary high valine is sufficient to induce NAFLD and accompanied by NAFLD-associated complications [33,34]. In common clinical practice treatment, dietary high-protein intervention that enriched in BCAA is presently the most useful treatment for NAFLD [[21], [22], [23]]. Limited understanding of the dietary protein or amino acids that ameliorate NAFLD has seriously obstructed the development of therapeutic drugs although recent studies have demonstrated that leucine and isoleucine can ameliorate hepatic steatosis [27], but the underlying mechanism remains elusive. Here, we demonstrate that, in genetically heterogeneous middle-aged laying hens, which better simulate the human population than hybrid strains, dietary supplementation of 67 % three BCAA alleviates NAFLD (Fig. 9). We found that High BCAA inhibited the degradation of BCKAs and the final products of propionyl-CoA and succinyl-CoA into the TCA cycle, leading to the accumulation of circulating BCAA and enhanced intermediates catabolism to drive increased ketogenesis. Furthermore, High BCAA intervention downregulates hepatic tryptophan metabolism and substantially results in hepatic and circulating tryptophan metabolite ILA decline, leading to AHR/MAPK9 mediated DNL downregulation. High BCAA feeding-induces MAPK9 ubiquitylation via inhibiting UFMylation mediated ubiquitin-proteasome autophagy activation, resulting in the ubiquitination degradation of DNL-associated proteins. Finally, High BCAA activates mitochondrial/peroxisomal fatty acid β-oxidation by activating PPAR-RXR and pexophagy to ameliorate hepatic steatosis and metabolic disorders, suggesting intake of dietary High BCAA as a new strategy to treat and prevent NAFLD and FLHS.

Each BCAA has different molecular and metabolic impacts although three BCAA are almost always combusted together and considered as a group, which may contribute to the differential sensing of each BCAA. For instance, a valine-specific catabolic intermediate 3-HIB regulates fatty acid *trans*-endothelial transportation and glucose uptake to drive glucotoxicity in skeletal muscle [98,99]. In contrast to previous reports that long-term intake of high BCAA diets induces obesity [9], whereas we found High BCAA feeding did not affect average body weight and body weight gain. In mammals, BCAA catabolism is first metabolized in most peripheral tissues, rather than the liver. The first catabolic step is reversible and all three BCAA are initially deamination by BCAT to yield glutamate and its respective BCKAs [100]. BCAT consists of BCAT1 and BCAT2, the former is localized to the cytosol, while the latter is present in the mitochondria. Nitrogen, derived from glutamate, can be transferred back to a BCKA by the BCAT enzymes and regenerate BCAA and α-KG [101]. However, Patrick and colleagues found that BCAT2 loss results in the accumulation of BCAA and BCKAs, but not BCAT1, increasing morbidity and mortality that can be alleviated by the restriction of dietary BCAA [35]. Mitochondrial BCAA metabolon formation mediated by BCAT2 and BCKDH is required for BCAT2 deficiency-induced phenotype, which can be reversed by BCAT1-mediated BCKAs reamination [35]. However, there is only one BCAT1 in the chicken (*Gallus gallus*) genome, indicating BCAT1 may play a unique role in different subcellular compartments (cytosol and mitochondria) and organs (tissue-specific and ubiquitously expressed) [[102], [103], [104], [105]]. These BCKAs are catabolized by BCKDC to produce corresponding intermediates and then undergo multiple additional steps after being processed by BCKDH to produce the end products that are specific to each BCAA: leucine is catabolized to produce acetoacetate and acetyl-CoA, glucogenic valine to produce propionyl-CoA, and isoleucine to produce both acetyl-CoA and propionyl-CoA [106]. In the present study, we used a middle-aged laying hens model fed a High BCAA diet and examined the catabolism of BCAA in peripheral tissue pectoralis and liver. Our study uncovered the catabolism of BCAA mainly occurred in the liver and that High BCAA intervention induced the inhibition of BCKAs degradation leads to the accumulation of circulating BCAA. The adverse metabolic effects of BCAA have been uncovered are mediated by isoleucine and valine, and the influence of isoleucine is stronger than valine [19]. However, our data indicate that increased circulating BCAA seems non-toxic and reprograms hepatic BCAA metabolism. We also found the final products of BCAA propionyl-CoA and succinyl-CoA are not into the TCA cycle but turn to increased ketogenesis that helps explain the unique metabolic roles of High BCAA and contribute to reprogramming hepatic lipid metabolism in a living organism.

Increased circulating BCAA levels in humans are associated with an increased risk of obesity [11,17], hepatic steatosis [7,107], IR [17,108] and T2D [109]. However, dietary protein supplementation does not alter plasma BCAA profiles [110] and a high-protein diet, enriched in BCAA, is the most useful approach for NAFLD treatment [[20], [21], [22]]. Multiple studies have highlighted long-term intake of high BCAA diets induces hyperphagia, obesity and decreased lifespan that is associated with tryptophan-mediated 5-HT reduction [9]. Similarly, our results indicate that intake of High BCAA leads to elevated circulating BCAA levels that seem not to be toxic; rather, the major adverse impacts of High BCAA under an isocaloric and iso-protein background, arise indirectly via hyperammonemia and increased reproductive hormones (data not shown). As a monoamine neurotransmitter that controls appetite, the reduction of 5-HT is of the essence in feeding behavior because tryptophan is the only precursor for 5-HT production [9]. Interestingly, we uncovered that tryptophan-mediated 5-HT depletion did not result in increased food intake and hyperphagia, suggesting moderately excess BCAA are not toxic although they decreased circulating 5-HT and melatonin. Melatonin and 5-HIAA as the downstream metabolites of 5-HT have been reported to affect food intake and metabolic disorder [9,111]. Melatonin is one of the hormones secreted by the pineal gland and widely involved in circadian rhythm entrainment and metabolic homeostasis [111]. 5-HIAA is decreased in T2D patients stool and supplementation with it can alleviate the pathogenesis of T2D [51]. Melatonin is also produced by the precursor 5-HT and whether the contribution of the pineal gland in the production of melatonin is stronger than 5-HT and the underlying mechanism between their interaction remains unknown.

Long-term intake of High BCAA diet results in declined AHR ligand ILA in the serum and liver, this widely reported tryptophan metabolite has been shown to enhance antitumor immunity via regulating CD8^+^ T cell and anti-inflammation [90,112]. The problem arises whether this rarely uncovered relationship between decreased circulating and hepatic ILA levels and High BCAA intake reflects intrinsic BCAA-AAA interaction or lipid metabolism. Recently, it has been identified that ILA is positively associated with T2D risk and promotes obesity [39,40]. Liver elimination and metabolism of endobiotics and xenobiotics (for instance, drugs, toxins, and overnutrition) are required for health [49]. Consistent with our present findings, a High BCAA diet significantly downregulated drug metabolism-cytochrome P450 and metabolism of xenobiotics by cytochrome P450 signaling pathways and accompanied by inhibited xenobiotic metabolism AHR signaling pathway, suggesting the major deleterious effects of excessive BCAA as xenobiotics are eliminated by restraining AHR signaling pathway. High BCAA diet-induced hepatic steatosis and DNL [9] are downregulated by AHR and MAPK9, which can be explained by the reduction of AHR ligand ILA in the circulation and the liver. Because ILA has been demonstrated to promote gut lipid absorption, enhance the uptake of fatty acid in WAT and increase fat mass [40]. Liver-specific knock-out AHR mice showed increased DNL activity, severe hepatic steatosis, inflammatory response and liver injury [50]. However, 5-HIAA derived from gut microbiota can improve glucose intolerance and obesity in HFD-fed mice, while preserving hepatic insulin sensitivity via directly activating AHR, which stimulates TSC2 transcription and thus inhibits mTORC1 signaling [51]. The MAPK signaling pathway is closely involved in the onset and development of obesity and IR [52], and inhibition of MAPKs is associated with less inflammatory cell infiltration, improved glucose tolerance, and ameliorated adipocyte cell diameter [53]. These results suggest that AHR/MAPK9 inhibited by High BCAA feeding is critical for hepatic DNL downregulation.

In addition to the AHR/MAPK9 signaling pathway, numerous signaling pathways likely mediated the metabolic advantages of a High BCAA diet. We also analyzed the contribution of other potential signaling pathways and examined the impacts of a High BCAA diet on amino acid sensing signaling pathways such as GCN2, mTORC1 and FGF19 signaling. The metabolism of leucine is glucose-dependent by leucyl-tRNA synthetase 1 (LARS1)-mediated shift in leucine utilization contributes to supporting cell survival under glucose deprivation [113]. The hepatic BCAA sensing is zonated, with leucine sensing by sestrin-mTORC1 in the liver controlling the response to dietary leucine [114]. This process is mediated by the 2.7 Å crystal structure of Sestrin2 in complex with leucine [[113], [114], [115]]. Previous reports suggest that the activity of hepatic GCN2 and mTORC1 is not necessary for the metabolic effects of a low isoleucine diet [19]. Consistently, we also found hepatic GCN2 and mTORC1 are not required for the metabolic effects of High BCAA feeding. These may be due to we may have initiated the High BCAA diet too long to activate GCN2 and mTORC1. Similarly, the improved hepatic insulin sensitivity and glucose homeostasis induced by High BCAA feeding is partly mediated by activating glycolysis or gluconeogenesis and inhibiting PI3K-Akt signaling pathway rather than activating or inhibiting hepatic GCN2 and mTORC1 signaling, or whole-body FGF19 signaling. *De novo* synthesized NEFA catalyzed by FASN is stored in LD in the form of TG to decrease lipotoxicity and serve as energy storage [116]. However, NEFA overload promotes DNL beyond liver capacity and induces intrahepatic TG accumulation and lipotoxicity, leading to hepatic steatosis and IR and then triggering a series of organelle dysfunction, cellular damage, inflammation, and metabolic disorders [2,3,117]. NAFLD induced by hepatic steatosis is commonly accompanied by decreased insulin sensitivity and glucose tolerance, which further develop from simple steatosis to NASH [2,5]. Herein, we demonstrate that High BCAA feeding not only downregulated DNL and decreased hepatic TG but also improved hepatic insulin sensitivity and glucose homeostasis and increased energy expenditure. On the other hand, we also found High BCAA feeding ameliorates NAFLD-related liver injury and fibrosis, ER stress, oxidative stress and ROS accumulation, highlighting the beneficial metabolic effects of High BCAA feeding on metabolic health. HNF4A and its target genes AGPAT2 and FASN also play critical functions in the metabolic impacts of hepatic lipid synthesis and LD formation [68,69]. Inversely, here, we suggest that supplementation of dietary BCAA by 67 % induces HNF4A transcription in the liver and downregulates the expression levels of its target genes AGPAT2 and PLIN2. There is no report suggesting that AHR/MAPK9 can impact or interact with HNF4A, which may be explained by the metabolic advantages of High BCAA feeding on AHR/MAPK9 and HNF4A are independent and suggest that the effects of High BCAA supplementation on host metabolism are systemic and widely.

Our most striking finding was that tryptophan-ILA depletion induced by High BCAA feeding led to AHR/MAPK9-mediated hepatic steatosis and DNL downregulation. Unlike the BCAA200 diet supplementation which contained twofold BCAA compared with the control BCAA100 diet) [9], 67 % High BCAA feeding decreases DNL not only in the liver but also does not impact appetite control. Long-term intake BCAA200 diet induces hyperphagia is associated with tryptophan-mediated 5-HT depletion and triggers hepatic steatosis and DNL [9], whereas we demonstrated that moderated elevation of dietary BCAA may have additional benefits for hepatic metabolism. Our results here hint that the hepatic reduction of LCFA and VLCFA mainly contribute to the inhibition of the AHR/MAPK9 signaling pathway induced by the High BCAA diet. We demonstrate that the lipogenesis reduction of High BCAA intake is not mediated by the activation of hepatic GCN2 and mTOR; rather, they contribute to inhibited DNL by AHR and MAPK9 and enhanced PPAR-RXR and pexophagy mediated fatty acid β-oxidation, perhaps in response to increased fatty acid intermediate metabolites carnitines. Another problem is why supplementation of High Leu, Ile and LeuIle has different impacts from elevation of BCAA. Our results demonstrated the toxic role of isoleucine [19] which severely damaged hepatocyte structure but was partly saved by High LeuIle, whereas we also observed a good tolerance of leucine in High Leu-fed hens. The downregulation of hepatic BCKAs catabolism and the end catabolic products of BCAA, along with the increase of BCAA catabolic intermediates, indicating that catabolism of BCAA is changed in High BCAA-fed hens. Understanding the function of leucine in the modulation of hepatic lipid metabolism, and illustrating the contribution of leucine from peripheral tissues to the elevated levels of circulating leucine we observed in High BCAA-fed hens, will be important to detail in the future.

It has been shown before that leucine and isoleucine alleviate obesity- and HFD-induced hepatic steatosis in mice by inducing PLIN2 ubiquitination degradation via activating UBR1/2 [27]. Comfortingly, we found that a High BCAA diet downregulated UFMylation, a ubiquitin-like post-translational protein modification by antagonizing ubiquitination, suggesting the activation of ubiquitination. We also observed activated proteasome and increased formation of autophagic vacuoles and numbers, demonstrating the activation of ubiquitin-proteasome autophagy. The ubiquitin-proteasome system (UPS) mediated ubiquitination plays a ubiquitous role in lipid metabolism, involving the uptake of fatty acid, DNL, and fatty acid β-oxidation [71]. Consistently, we also found High BCAA feeding-induced profound changes in ubiquitylation sites of the DNL, lipolysis, and fatty acid oxidation-related proteins. ACLY, ACC, FASN, and SCD1 are all targets for ubiquitination. The ubiquitination and degradation of ACC, FASN and ACLY are beneficial for the suppression of DNL and contribute to enhanced fatty acid oxidation, whereas their deubiquitination enhances TG accumulation [71]. Alongside fatty acid synthesis, the UPS also controls fatty acid desaturation mediated by SCD1, a critical enzyme that catalyzes the production of MUFA. Herein, we identified High BCAA intervention induces extensive ubiquitination in ACLY, ACC, FASN, and SCD, revealing these lipogenesis key proteins not only remodeling in transcription but also modification in post-translation. In addition, we also observed increased ubiquitination in fatty acid elongation proteins such as ACSL1, ACACA, and ACSBG2, which is in line with the reduction of LCFA, VLCFA, UFA, PUFA and MUFA. UPS mediated PLIN2 ubiquitination and deubiquitination of ATGL and VCP thereby restraining LD formation and accelerating TG hydrolysis, which is confirmed by our results. UPS plays a critical role in controlling fatty acid β-oxidation., For instance, the enzymes of HSD17B4 and CPT2 are essential in fatty acid oxidation and transport into mitochondria [71]. Our results indicate that High BCAA suppresses the polyubiquitination activation of fatty oxidation related-proteins including CPT1A, ACOX1, ACAA1, EHHADH, HADHA, HMGCS2, ALDH1A1 and ALDH7A1. In summary, these results suggest that ubiquitination degradation of DNL, fatty acid elongation and LD formation-related proteins mediated by UPS and the deubiquitination of lipolysis and fatty acid oxidation-related proteins contribute to the inhibition of DNL and the catabolism of fatty acids, which is necessary for the amelioration of NAFLD. However, we observed the deubiquitination of AHR is increased, whereas the polyubiquitination of MAPK9 at K265, K250 and K153 sites was activated, which can be explained by AHR being directly inhibited by tryptophan-ILA depletion and MAPK9 being indirectly downregulated by ubiquitination. Clarifying both the AHR-direct and MAPK9-indirect mechanisms by which an increase in different dietary BCAA levels promotes hepatic lipid metabolism will likewise be an interesting area of future research. Future studies will be asked to define how AHR/MAPK9 post-translational modification is changed by High BCAA intervention and to clarify the function of ubiquitination in the metabolic response to increased dietary BCAA.

Low isoleucine and low amino acid diets both increased energy expenditure by inducing the FGF21-UCP1 axis [19], we also found increased oxidative phosphorylation, pentose phosphate pathway, and elevated hepatic and circulating ATP, uncovering the increase of energy expenditure. However, we observed no giant variations in either circulating FGF19, ileum or hepatic FGF19 mediated by either High BCAA diet, and suggest that the advantages metabolic effects of High BCAA feeding are FGF19 independent. As an important transcription factor, Krüppel-like factor 15 (KLF15) takes part in controlling glycemic, lipid, and amino acid metabolism of the host, in particular in BCAA metabolism [118]. For instance, KLF15 expression was impressed by BCAA abundance while BCAA starvation stimulated KLF15 expression [119]. Intriguingly, herein, we found a High BCAA diet upregulated hepatic KLF15 expression, suggestive of a unique response mechanism of 67 % BCAA supplementation, perhaps in response to the elimination of xenobiotics overnutrition. Elucidating the function of KLF15 in the control of hepatic lipid metabolism mediated by High BCAA, and illustrating the contribution of KLF15 from circulation to the upregulated hepatic KLF15 we found in High BCAA-fed hens, will be interesting to address in future research. Furthermore, we elucidated High BCAA feeding induces the activation of PPAR-RXR mediated fatty acid β-oxidation and ketogenesis, reprogramming hepatic lipid profile. Nonetheless, it is worth pointing out that mitochondria are not only important for fatty acid β-oxidation, but also is required for the catabolism of BCAA. As previous mentioned that the formation of a mitochondrial BCAA metabolon mediated by BCAT1 and BCKDH in controlling BCAA catabolism plays a critical role [35]. Yoneshiro and colleagues reported that BAT actively utilizes BCAA in the mitochondria for thermogenesis via SLC25A44 mediated BCAA transportation into mitochondria and promotes systemic BCAA clearance in mice and humans under cold exposure [97]. Consistently, they also found that mitochondrial BCAA import via SLC25A44 is required for BCAA-derived metabolites non-essential amino acids and glutathione synthesis, which contributes to ameliorating oxidative stress and IR and is independent of thermogenesis [120]. However, because the chicken genome lacks SLC25A44 and therefore identifying the mitochondria BCAA carrier in laying hens will likewise be a key area of future study. In the present study, we suggest that High BCAA feeding improved cellular mitochondria morphology and structure, this is in alignment with activated fatty acid β-oxidation and increased fatty acyl-carnitines, uncovering the activation of mitochondrial beneficial function in BCAA and lipid catabolism. As a group of transcription factors, peroxisome proliferator-activated receptors (PPARs) regulate fatty acid metabolism at a transcriptional level. The UPS is necessary for fine-tuning PPARγ activity via facilitating ubiquitination of its ligand-binding domain upon ligand binding, thus acting as a negative feedback loop that impresses the transcriptional activity of PPARγ [77]. We identified the activation of the PPAR pathway induced by High BCAA feeding not only at the transcriptional level but also at the post-translational protein level, which promotes fatty acid oxidation via activating gene transcription and inhibiting protein ubiquitination. Further study will be needed to systematically define the structural basis of heterodimerization of PPAR with RXR stimulated by high BCAA [71,77], and uncover both the PPAR-RXR-dependent and PPAR-RXR-independent mechanisms by which a High BCAA diet promotes metabolic health will likewise be an interesting topic of future research.

Pexophagy has been strongly associated with the β-oxidation of VLCFA and is also important for ROS detoxification [80]. Consistent with our current findings in middle-aged hens, a High BCAA diet activated pexophagy. These data are consistent with our present findings that the reduction of hepatic VLCFA and ROS induced by High BCAA feeding in middle-aged hens, is most likely due to peroxisomal biogenesis stimulated by a High BCAA diet. A recent report demonstrates that peroxisomal β-oxidation derived acetyl-CoA inhibits lipophagy by inducing the activation of mTORC1 [84]. However, we found that High BCAA did not activate mTORC1, and we may have initiated the High BCAA diet too long to stimulate mTORC1. The function of peroxisomes in metabolic health is intricated; because peroxisomes are critical metabolic organelles, famous for their functions in the metabolism of complex lipids and ROS [121]. It has been suggested that peroxisomes also act as central regulators of lipolysis via recruiting and activating the lipase ATGL [82,83]. This is in alignment with present results that enhanced hepatic lipolysis and increased deubiquitination of ATGL and VCP. In the cytosol, protein is recognized by the soluble receptor PEX5 and imported into peroxisomes, and that PEX5 must then return to the cytosol, which recycling results in PEX5 being monoubiquitinated by the PEX2-10-12 ubiquitin ligase complex and then monoubiquitinated PEX5 is subsequently extracted from the matrix into the cytosol via the PEX1-PEX6 ATPase complex [78]. It is conceivable that the activation of ubiquitin-proteasome autophagy induced by High BCAA feeding in the liver not only promotes MAPK9 ubiquitination degradation but also triggers PPAR-RXR and pexophagy activation.

However, several disadvantages of our research are acknowledged. Our study was performed completely in laying hens, whereas recent studies have observed sex-dependent differences in the response to dietary BCAA [9,18]. Furthermore, our study analyzed only a 67 % dietary High BCAA intervention, and researches on BCAA as well as protein intervention demonstrates that different strains and sexes may respond differentially to various levels of BCAA supplementation [9,[122], [123], [124]]. Different concentrations of BCAA intervention may more effectively function to ameliorate hepatic steatosis in laying hens, and analyzing the different responses to different levels of BCAA may provide new insights into both physiological mechanisms and clinical transformation. The impact of the different concentrations of BCAA intervention on the NAFLD of various ages and both sexes should be considered in future research. Third, the basal diets used in the present study were provided by a combination of vegetable protein and synthetic essential amino acids, which is different from a combination of casein and other amino acids [9,19,20]. The dietary composition difference may affect palatability, food consumption and metabolic impact. Last but not the only, our study did not directly measure BCAA turnover and catabolism in peripheral tissues; thus, the destiny of excessive dietary BCAA and whether they help directly to alleviate NAFLD and IR remains unclear. We have not yet identified the beneficial effects of High BCAA intervention in mammals and humans with NAFLD whether according to in laying hens due to physiological differences across species, and this is an important area for future study.

In summary, dietary High BCAA intervention is an important modulator of amelioration of FLHS in middle-aged laying hens and potentially NAFLD in humans. Surprisingly, intake of 67 % High BCAA ameliorates NAFLD by inhibiting tryptophan-ILA-AHR and activating MAPK9 ubiquitination, inhibiting DNL and steatosis, increasing hepatic insulin sensitivity, promoting ketogenesis and fatty acid oxidation via activating PPAR-RXR and pexophagy. Our results illustrate unique mitigative roles for the dietary High BCAA in NAFLD and highlight the critical role of microautophagy associated pexophagy and ubiquitin-proteasome autophagy for lipid metabolism. Finally, our results suggest that intake of 67 % dietary High BCAA may be a new treatment and public health strategy to resist the twin epidemics of FLHS and NAFLD.

## Funding statement

This work was supported by the Earmarked Fund for Modern Agro-Industry Technology Research System of China (CARS-40-K10) and the twinning service plan of Zhejiang provincial team science and technology special commissioner of China and the science and technology development project of Hangzhou [202003A02].

## CRediT authorship contribution statement

**Huafeng Jian:** Writing – review & editing, Writing – original draft, Visualization, Methodology, Investigation, Data curation, Conceptualization. **Ru Li:** Writing – original draft, Methodology, Data curation. **Xuan Huang:** Software, Methodology, Investigation. **Jiankui Li:** Methodology, Investigation, Data curation. **Yan Li:** Methodology, Data curation. **Jiangang Ma:** Writing – review & editing, Software. **Mingkun Zhu:** Writing – review & editing, Software, Resources. **Xinyang Dong:** Writing – review & editing, Writing – original draft, Funding acquisition, Conceptualization. **Hua Yang:** Writing – review & editing. **Xiaoting Zou:** Writing – review & editing, Writing – original draft, Supervision, Project administration, Funding acquisition, Conceptualization.

## Declaration of competing interest

The authors declare that they have no known competing financial interests or personal relationships that could have appeared to influence the work reported in this paper.

## Data Availability

No data was used for the research described in the article.
