# Supplementary material for: Branched-chain amino acids alleviate NAFLD via inhibiting de novo lipogenesis and activating fatty acid β-oxidation in laying hens
Source: Redox Biol. 2024 Oct 3;77:103385. doi: 10.1016/j.redox.2024.103385 (PMC11536022; doi:10.1016/j.redox.2024.103385)
Supplement: Multimedia component 1 [file mmc1.docx]

**Supplementary data**

**Branched-chain amino acids alleviate NAFLD via inhibiting de novo lipogenesis and activating fatty acid β-oxidation in laying hens**

**This file includes:**

Supplementary Fig. S1 to S12

Supplementary Table S1 to S3

Table S1 Requirement and excess of branched-chain amino acids in commercial laying hens during the laying peak period

| Item | Leucine (%) | Isoleucine (%) | Valine (%) | Productive performance | Reference |
| --- | --- | --- | --- | --- | --- |
| NRC (1994) (26-52wk) | 0.82 | 0.65 | 0.70 | Recommended optimum requirement | [1] |
| NY/T33-2004 (26-52wk) | 0.98, 1.02 | 0.66, 0.72 | 0.54, 0.59 | Recommended optimum requirement | [2] |
| Fengda No.1 (26-52wk) | 1.20 | 0.65 | 0.70 | Improved laying rate, 0.79 is adverse | [3] |
| Hy-Line W-36 (28-34wk) | 1.37, 1.38 | 0.37, 0.50, 0.64, 0.78, 0.92 | 0.47, 0.61, 0.74, 0.88, 1.02 | The optimum for maximum egg mass were isoleucine 0.79 and valine 0.93 | [4] |
| Hy-Line Brown (40-47wk) | 1.30 | 0.67 | 0.69, 0.79, 0.89, 099, 1.09 | Not affect productive performance and egg quality | [5] |
| Lohmann Brown (28-40wk) | 0.82 | 0.54, 0.64, 0.74, 0.84, 0.94 | 0.70 | Not affect productive performance and egg quality | [6] |
| Hy-Line W-36 (41-60wk) | 1.02 | 0.66 | 0.515, 0.585, 0.655, 0.725, 0.795, 0.865 | The optimum valine for highest egg mass was 0.725 and no adverse impact | [7] |
| Lohmann Brown (25-28wk) | 1.01, 1.15 | 0.57, 0.80, 1.15 | 0.63, 0.72 | Deceased daily egg mass, feed intake and body weight gain | [8] |
| Lohmann Brown (25-32wk) | 0.79 | 0.39, 0.45, 0.51, 0.57, 0.69, 0.75, 0.81 | 0.44, 0.50, 0.56, 0.62, 0.68, 0.74, 0.80, 0.86 | Deceased BW and daily egg mass when isoleucine higher than 0.8 and 1.00-1.15, respectively. Optimum valine for daily egg mass at 24-32w was 0.615, at 25-32w was 0.685, at 46-52w was 0.585. 1.06 valine no adverse impact, 1.36 valine reduced feed consumption and daily egg mass | [9] |
| Lohmann Brown (24-32wk) | 0.78 | 0.37, 0.40, 0.43, 0.46, 0.51, 0.57, 0.81, 1.05 | 0.51, 0.56, 0.61, 0.66, 0.71, 0.76, 1.06, 1.36 |  |  |
| Lohmann Brown (29-30wk) | 0.78 | 0.58 | 0.51, 0.56, 0.61, 0.66, 0.71, 0.76, 1.06, 1.36 |  |  |
| Lohmann Brown (46-54wk) | 0.78 | 0.58 | 0.51, 0.56, 0.61, 0.66, 0.71, 0.76 |  |  |
| Hy-Line W-36 (41-46wk) | 0.82 | 0.519, 0.550, 0.580, 0.588, 0.610, 0.616, 0.671 | 0.525, 0.560, 0.595, 0.630, 0.665, 0.700, 0.765 | The optimum valine for egg mass was 0.630 and no adverse impact | [10] |

Table S2 Composition and nutrient levels of the basal diet (air-dry basis) ^a^.

| Amino Acid Defined Diets | Ctrl AA | High Leu | High Ile | High LeuIle | High BCAA |
| --- | --- | --- | --- | --- | --- |
| Diet name | 14.70% protein | Leu 2/3 Add, 14.70% protein | Ile 2/3 Add, 14.70% protein | LeuIle 2/3 Add, 14.70% protein | BCAA 2/3 Add, 14.70% protein |
| Color | Black | Gray | Orange | Yellow | Blue |
| Formula | g/100g | g/100g | g/100g | g/100g | g/100g |
| Corn | 67.00 | 67.00 | 68.00 | 68.06 | 68.10 |
| Soybean meal | 10.50 | 11.81 | 11.50 | 13.95 | 15.86 |
| Wheat bran | 2.00 | 1.90 | 0.90 | 1.00 | 1.45 |
| Peanut meal | 6.77 | 4.00 | 4.68 | 1.10 | 0.00 |
| Rapeseed meal | 1.80 | 1.80 | 2.40 | 2.00 | 0.00 |
| Limestone | 9.30 | 9.28 | 9.28 | 9.25 | 9.25 |
| Soybean oil | 0.40 | 1.00 | 0.50 | 0.90 | 1.10 |
| Methionine (98%) | 0.14 | 0.15 | 0.14 | 0.15 | 0.15 |
| Lysine (78.8%) | 0.11 | 0.12 | 0.11 | 0.10 | 0.08 |
| Threonine | 0.00 | 0.00 | 0.00 | 0.00 | 0.00 |
| Tryptophan | 0.00 | 0.00 | 0.00 | 0.00 | 0.00 |
| Arginine (99%) | 0.05 | 0.14 | 0.11 | 0.21 | 0.23 |
| Valine (99%) | 0.11 | 0.12 | 0.11 | 0.12 | 0.60 |
| Leucine (99%) | 0.00 | 0.83 | 0.00 | 0.83 | 0.83 |
| Isoleucine (99%) | 0.17 | 0.17 | 0.61 | 0.61 | 0.61 |
| CaHPO4 | 0.60 | 0.63 | 0.62 | 0.68 | 0.70 |
| Salt | 0.36 | 0.36 | 0.36 | 0.36 | 0.36 |
| Choline chloride (60%) | 0.21 | 0.21 | 0.21 | 0.21 | 0.21 |
| Mineral and vitamin premix^b^ | 0.48 | 0.48 | 0.48 | 0.48 | 0.48 |
| Calculated nutritional level, % | | | | | |
| Metabolizable energy (ME), MJ/Kg | 2.68 | 2.68 | 2.68 | 2.68 | 2.68 |
| Calcium | 3.59 | 3.59 | 3.59 | 3.59 | 3.59 |
| Analyzed nutritional level, % | | | | | |
| Crude protein (CP) | 14.68 | 14.70 | 14.71 | 14.72 | 14.73 |
| Total phosphorus | 0.46 | 0.46 | 0.46 | 0.46 | 0.46 |
| Methionine | 0.36 | 0.36 | 0.36 | 0.36 | 0.36 |
| Lysine | 0.66 | 0.66 | 0.66 | 0.66 | 0.66 |
| Threonine | 0.48 | 0.48 | 0.48 | 0.48 | 0.48 |
| Tryptophan | 0.14 | 0.14 | 0.14 | 0.14 | 0.14 |
| Arginine | 1.03 | 1.03 | 1.03 | 1.03 | 1.03 |
| Leucine | 1.20 | 2.00 | 1.20 | 2.00 | 2.00 |
| Isoleucine | 0.65 | 0.65 | 1.08 | 1.08 | 1.08 |
| Valine | 0.70 | 0.70 | 0.70 | 0.70 | 1.17 |

^a^Analyzed value of pooled experimental diets Ctrl AA, High Leu, High Ile, High LeuIle, and High BCAA %. ^b^The premix provided following per kilogram of diet: vitamin A, 7,500 IU; vitamin D3, 2500 IU; vitamin E, 49.5 mg; vitamin K3, 2.5 mg; vitamin B1, 1.5 mg; vitamin B2, 4 mg; vitamin B6, 2 mg; vitamin B12, 0.02 mg; niacin, 30 mg; folic acid, 1.1 mg; pantothenic acid, 10 mg; biotin,0.16 mg; chloride choline, 400 mg; Fe, 80 mg; Cu, 20 mg; Mn, 60 mg; Zn, 80 mg; I, 0.8 mg.

Table S3. Primers used for quantitative real-time PCR.

| Gene | Primer | Primer Sequence (5'-3') | GenBank number |
| --- | --- | --- | --- |
| β-Actin | Forward | TCCCTGGAGAAGAGCTATGAA | NM_205518.1 |
|  | Reverse | CAGGACTCCATACCCAAGAAAG |  |
| PepT1 | Forward | CTTGGCAGATCCCTCAGTATTT | XM_034074354.1 |
|  | Reverse | GTTGGGCTTCAACCTCATTTG |  |
| B^0^AT1 | Forward | CATGATCGGACACAAGCCCA | XM_419056.6 |
|  | Reverse | AGCATAGACCCAGCCAGGATA |  |
| ATB^0,+^ | Forward | TAAACCAGTGCAATTTCCCA | XM_001199603.1 |
|  | Reverse | CGATGTTGCCAGTCTCATC |  |
| LAT1 | Forward | TGGCCTTGTAC AGTGGTCTT | NM_001030579.2 |
|  | Reverse | GCTTCGGACTTC AGC ATCTG |  |
| LAT4 | Forward | ACAACTGTGGGACGCCGACTGA | XM_415803.6 |
|  | Reverse | GGCATTGGTGGCATTGGTGATTT |  |
| SLC38A9 | Forward | ACTTTCCCAGCAACGA | XM_046905524 |
|  | Reverse | GATGAGCGACGGATACA |  |
| B^0^AT3 | Forward | CCGTTGTGGAGACTGAATG | XM_003640787.5 |
|  | Reverse | CCCGCACCAAGAAGCA |  |
| BCAT1 | Forward | GGAGGATGTTCAGAGGA | XM_416424.7 |
|  | Reverse | TGGCAGCAGTTGAGTAG |  |
| BCKDHA | Forward | AACTTCGCCGCCACCCT | XM_025144505.3 |
|  | Reverse | CCTGTTTGTCCCAGTAGTTCACC |  |
| BCKDHB | Forward | TATCGTTACCGCTCTGG | NM_204657.2 |
|  | Reverse | ATCGCTGCCTTGTCG |  |
| BCKDK | Forward | GGCTGTTGCGTCCCTTCCTG | XM_040694086.2 |
|  | Reverse | AAGTCGGGCTTGTCCTCGTGTAG |  |
| DBT | Forward | AGGGCATTACAGAGGTG | NM_204656.2 |
|  | Reverse | AGCCAGACGACGAACT |  |
| DLD | Forward | AGGCTCAGAAGTTGCTCCC | NM_001030727.3 |
|  | Reverse | GCCAAACCGAACCCAGT |  |
| HIBADH | Forward | CTGCCTTCAAGTCCCA | NM_001006362.3 |
|  | Reverse | TCCATTCCACCAACCA |  |
| HIBCH | Forward | CAGACCCAAGGTTCTCAAT | NM_001031243.2 |
|  | Reverse | GCCGCCAGCACAAA |  |
| IVD | Forward | TGCTGGGTCTGATGTTGTG | NM_001199561.2 |
|  | Reverse | GCTTCTGTGCCGTGCTAAA |  |
| MCCC1 | Forward | GGCGTTCCTGTTGTTG | XM_046923902.1 |
|  | Reverse | ATGCCCTTTCCTCCA |  |
| MCCC2 | Forward | GAAAGCCCTGGTAACTC | XM_046937242.1 |
|  | Reverse | CAAGAACGGAGACCCT |  |
| PPM1K | Forward | AGACCGCTTTGATTACGC | XM_420574.8 |
|  | Reverse | CCCACGCTTGCCACTA |  |
| HADH | Forward | CTTCAACCCTGTGCCT | NM_001277897.2 |
|  | Reverse | CCAACCCAACATAGTCC |  |
| HADHB | Forward | GGCGTCCGTATTCCGTTCT | NM_001389485.2 |
|  | Reverse | TTGGCTCGGTTCAGGGTG |  |
| PLIN2 | Forward | GTTGCCAATGCTAAGGGTGT | NM_001031420.2 |
|  | Reverse | ACCACACGACTTCCCAAGAC |  |
| UBR1 | Forward | CGACGGCAACGAGA | XM_015287098.4 |
|  | Reverse | GAACACCTTCCCACAGA |  |
| UBR2 | Forward | ATAACCCGTCAAGTAGGA | XM_419446.8 |
|  | Reverse | TGGTCTCAACCGAATGT |  |
| ACAA1 | Forward | CCTTTGCCTTGGCTTCC | NM_001197288.2 |
|  | Reverse | GCTGACCTGACTGGCGTTA |  |
| ALDH9A1 | Forward | GCCGAGTGATAACCAAGC | XM_422248.8 |
|  | Reverse | CAGGCTCCAATCCCAAC |  |
| AOX1 | Forward | GGCATTGGACTTTACAC | NM_001038692.2 |
|  | Reverse | TTCAGCAGTCAGAGGG |  |
| EHHADH | Forward | TCTGCGTGAGTACAGGGAC | XM_422690.8 |
|  | Reverse | GGGAACATCAGGGTGGA |  |
| HADHA | Forward | ATCTTCGCCAGTAACACG | NM_205056.3 |
|  | Reverse | GTAGAAACCAGGTCCATCC |  |
| HMGCS2 | Forward | ATCAAGCGGCCTTTCACC | XM_422225.8 |
|  | Reverse | CAGGGAGGGTTTGGTCTTCT |  |
| PCCA | Forward | TGTGGCTGAACAACTGAG | XM_040706028.2 |
|  | Reverse | GATGGACAACAAGGGTG |  |
| PLIN1 | Forward | TGCTGCTTGTTGAAGAACCACT | NM_001127439.1 |
|  | Reverse | AGGCATTCTGTGATGATTATGTGGT |  |
| FABP3 | Forward | GACAAGGTGACGGTGAAGA | NM_001030889.2 |
|  | Reverse | ATAGGTGCGGGTGGAGA |  |
| ACACA | Forward | GCTGCTGAGGAAGTTGG | NM_205505.2 |
|  | Reverse | TGATGCCTGCGTTGC |  |
| APOA4 | Forward | GCTGAAGGAGCAGGTGGCA | NM_204938.3 |
|  | Reverse | CGGAAGGTCTCGCTGATGG |  |
| APOA5 | Forward | ACGGAGTGGCTTCTGGGAGT | XM_417939.8 |
|  | Reverse | GGCTGTCGGAGTCGTGGTA |  |
| APOA1 | Forward | GTGACCCTCGCTGTGCTCTT | NM_205525.5 |
|  | Reverse | CACTCAGCGTGTCCAGGTTGT |  |
| FAAH | Forward | ATCACGGAGTACATGCAGGAAAGC | XM_422450.7 |
|  | Reverse | TGCCCAAAGATACCAGAAACGAC |  |
| ELOVL2 | Forward | TGGACCCACTTTGAATAGC | NM_001197308.2 |
|  | Reverse | CGCATAGAAGGGATGACTG |  |
| ELOVL3 | Forward | GACCTATCGCACCTGGAAG | NM_001318410.2 |
|  | Reverse | AAGCCCGCTGCTCGTA |  |
| ELOVL5 | Forward | GCCTTGTGGTCACTCTTAT | NM_001199197.2 |
|  | Reverse | GAGGACGCCTTCTTGTT |  |
| SLC6A4 | Forward | GCAACGAGCAATGAGAC | XM_015295699.4 |
|  | Reverse | GAGCCGTAGGGCAGAT |  |
| ACMSD | Forward | TGGGCGAAACCTCA | XM_040704311.2 |
|  | Reverse | TTCAGCGGCAGCAT |  |
| HAAO | Forward | GCCCTTCCCTCTAAAT | XM_040667561.2 |
|  | Reverse | CTCCAAACACGCTCAA |  |
| CAT | Forward | GTTGGCGGTAGGAGTCTGGTCT | NM_001031215 |
|  | Reverse | GTGGTCAAGGCATCTGGCTTCTG |  |
| SOD1 | Forward | TTGTCTGATGGAGATCATGGCTTC | NM_205064.2 |
|  | Reverse | TGCTTGCCTTCAGGATTAAAGTGAG |  |
| AGPAT2 | Forward | GCCGCACCGTCAAGAATA | XM_001235299.7 |
|  | Reverse | ACCTCCGCCATCACCATC |  |
| HNF4A | Forward | ACCGAATCAGCACCCG | XM_025142195.3 |
|  | Reverse | GACCTCTTGGCAACTCCC |  |
| PEX5 | Forward | CCTTGGCTTACTGATTACGA | NM_001012818.3 |
|  | Reverse | TGGGCATAGGCTGGTTTA |  |
| PEX6 | Forward | GCAGACACCCAAAA | XM_015283550.4 |
|  | Reverse | CCGAAGAGCATCAC |  |
| LOC107049500 (PEX19) | Forward | GCCAAGAAATCCCC | XM_015273162.4 |
|  | Reverse | CCAATTCCCCATCA |  |
| ACBD3 | Forward | CTGCCAAGAAATCC | XM_046938767.1 |
|  | Reverse | TTCCCCATCAAACA |  |
| ACBD4 | Forward | CCGAGATGAAGAAGG | XM_040692302.2 |
|  | Reverse | TGGTATCGCAGAACA |  |
| FASN | Forward | CCTGGAGATGTGGAGTATGTTG | NM_205155.3 |
|  | Reverse | TCAAGGAGCCATCGTGTAAAG |  |
| SCD1 | Forward | CCTTGCGATACGTCTGGAGG | XM_005025224.1 |
|  | Reverse | CGAAACACAGAACGGCCCA |  |
| GLUT4 | Forward | CTCTGTCGGTCGCCATCTTCT | XM_046906205.1 |
|  | Reverse | GTGGAATCCCATCCTGTGCC |  |
| CD36 | Forward | ACTGCGCTTCTTCTCCTCTGA | NM_001030731.1 |
|  | Reverse | TCACGGTCTTACTGGTCTGGTAAA |  |
| FABP1 | Forward | GCAGAATGGGAATAAGTT | NM_204192.4 |
|  | Reverse | TTGTATGGGTGATGGTGT |  |
| FATP1 | Forward | TACAATGTGCTCCAGAAGGG | NM_001039602.2 |
|  | Reverse | GTCTGGTTGAGGATGTGACTC |  |

**References**

[1] G.M. Pesti, Nutrient requirements of poultry. Animal Feed Science and Technology. 56(1995)1177-178.

[2] S. Jiang, H.K. El-Senousey, Q. Fan, X. Lin, Z. Gou, L. Li, Y. Wang, A.M. Fouad, Z. Jiang, Effects of dietary threonine supplementation on productivity and expression of genes related to protein deposition and amino acid transportation in breeder hens of yellow-feathered chicken and their offspring. Poult Sci. 98(2019)126826-6836.

[3] H. Jian, S. Miao, Y. Liu, H. Li, W. Zhou, X. Wang, X. Dong, X. Zou, Effects of dietary valine levels on production performance, egg quality, antioxidant capacity, immunity, and intestinal amino acid absorption of laying hens during the peak lay period. Animals (Basel). 11(2021)7.

[4] K. Bregendahl, S.A. Roberts, B. Kerr, D. Hoehler, Ideal ratios of isoleucine, methionine, methionine plus cystine, threonine, tryptophan, and valine relative to lysine for white leghorn-type laying hens of twenty-eight to thirty-four weeks of age. Poult Sci. 87(2008)4744-758.

[5] M.M.M. Azzam, X.Y. Dong, L. Dai, X.T. Zou, Effect of excess dietary l-valine on laying hen performance, egg quality, serum free amino acids, immune function and antioxidant enzyme activity. Br Poult Sci. 56(2015)172-78.

[6] X.Y. Dong, M.M.M. Azzam, X.T. Zou, Effects of dietary l-isoleucine on laying performance and immunomodulation of laying hens. Poult Sci. 95(2016)102297-2305.

[7] J. Wen, A. Helmbrecht, M.A. Elliot, J. Thomson, M.E. Persia, Evaluation of the valine requirement of small-framed first cycle laying hens. Poult Sci. 98(2019)31272-1279.

[8] S. Peganova,K. Eder, Interactions of various supplies of isoleucine, valine, leucine and tryptophan on the performance of laying hens. Poult Sci. 82(2003)1100-105.

[9] S. Peganova,K. Eder, Studies on requirement and excess of valine in laying hens. Archiv Fur Geflugelkunde. 66(2002)241-250.

[10] R.H. Harms,G.B. Russell, Evaluation of valine requirement of the commercial layer using a corn-soybean meal basal diet. Poult Sci. 80(2001)2215-218.


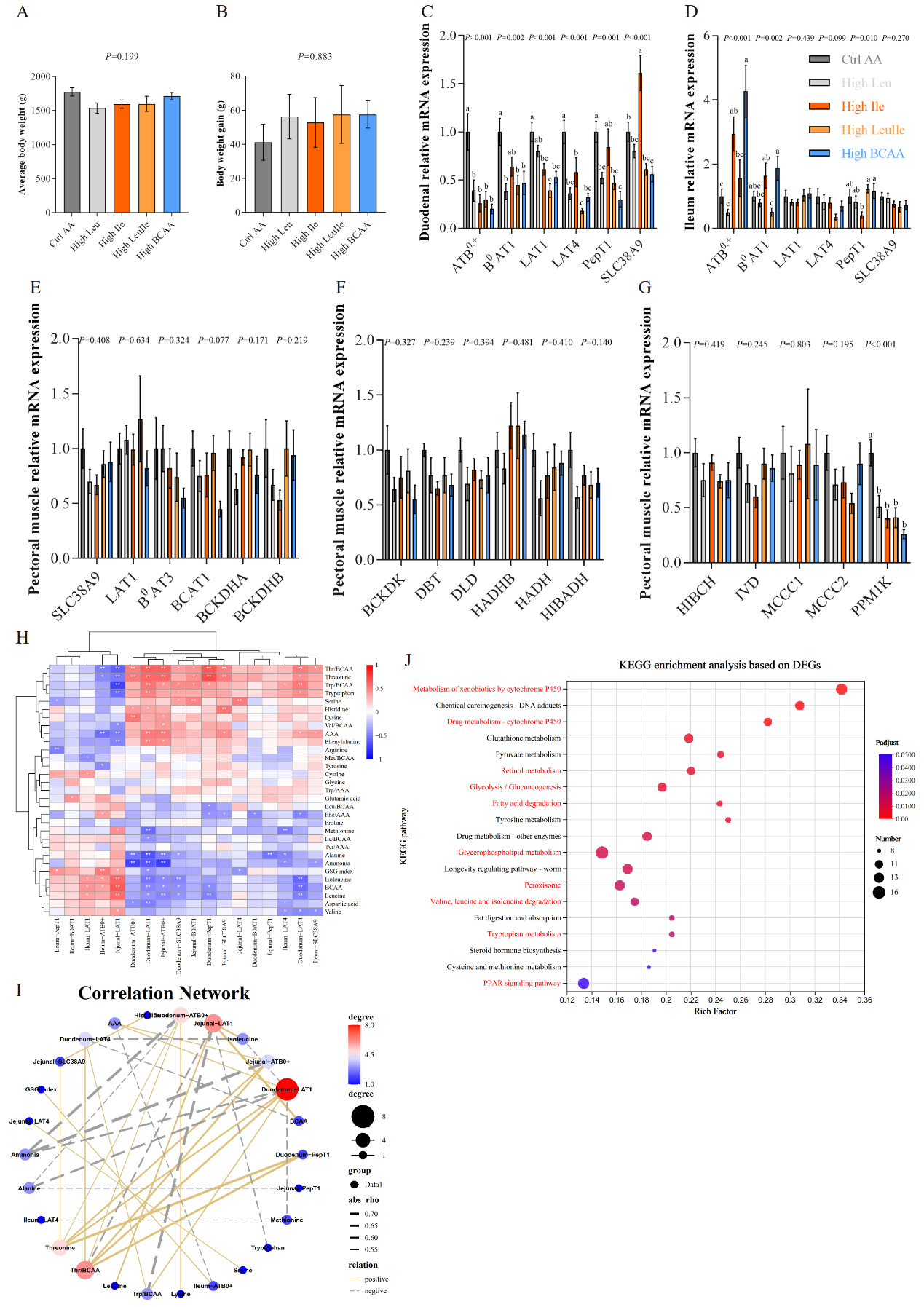


**Fig. S1, related to Fig. 1. High BCAA diet reprograms hepatic BCAA metabolism (n=5-6/group).** (A-B) Body weight and body weight gain of laying hens during feeding. (C-D) mRNA expression of amino acid transporters in duodenum and ileum. (E-G) mRNA expression of amino acid transporters and BCAA catabolism associated genes in pectoral muscle. (H-I) Correlation heatmap and network analysis of intestinal amino acid transporters expression and serum free amino acids. (J) KEGG diagram enrichment analysis of DEGs in the liver of Ctrl AA and High BCAA-fed laying hens.


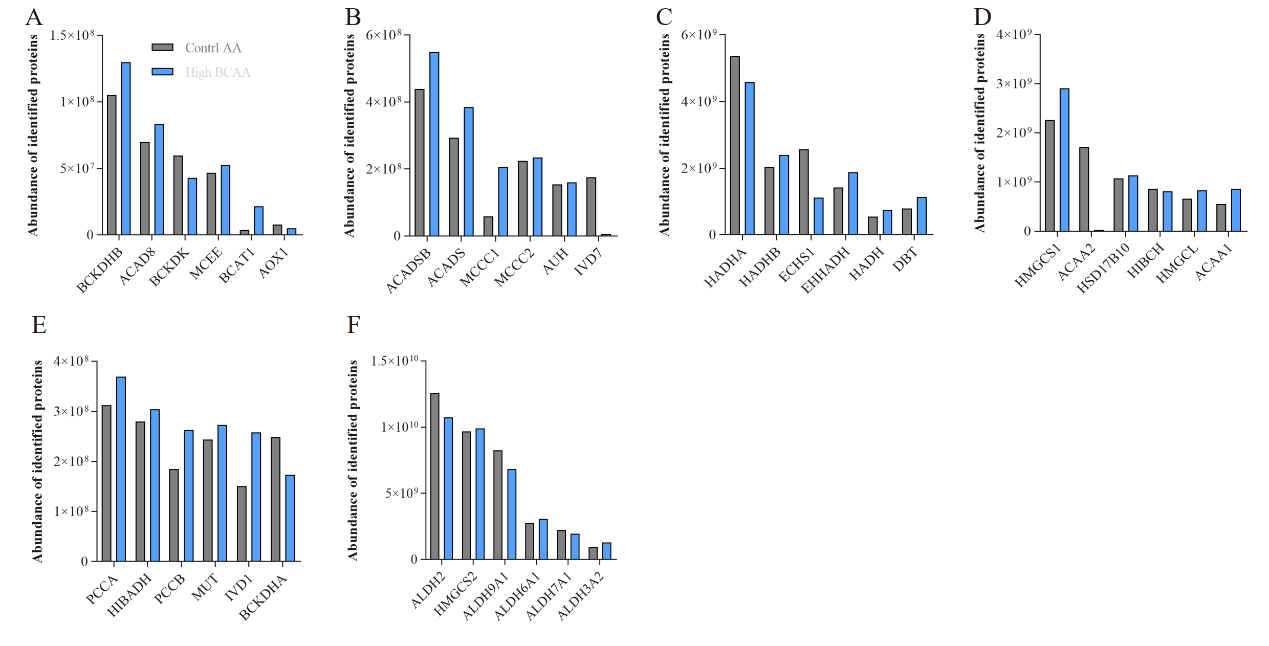


**Fig. S2, related to Fig. 1. Effects of High BCAA diet on hepatic BCAA catabolic proteins (n=2/group).** (A-F) Identified protein abundance of BCAA catabolism in the livers of Ctrl AA and High BCAA-fed laying hens (n=2/group).


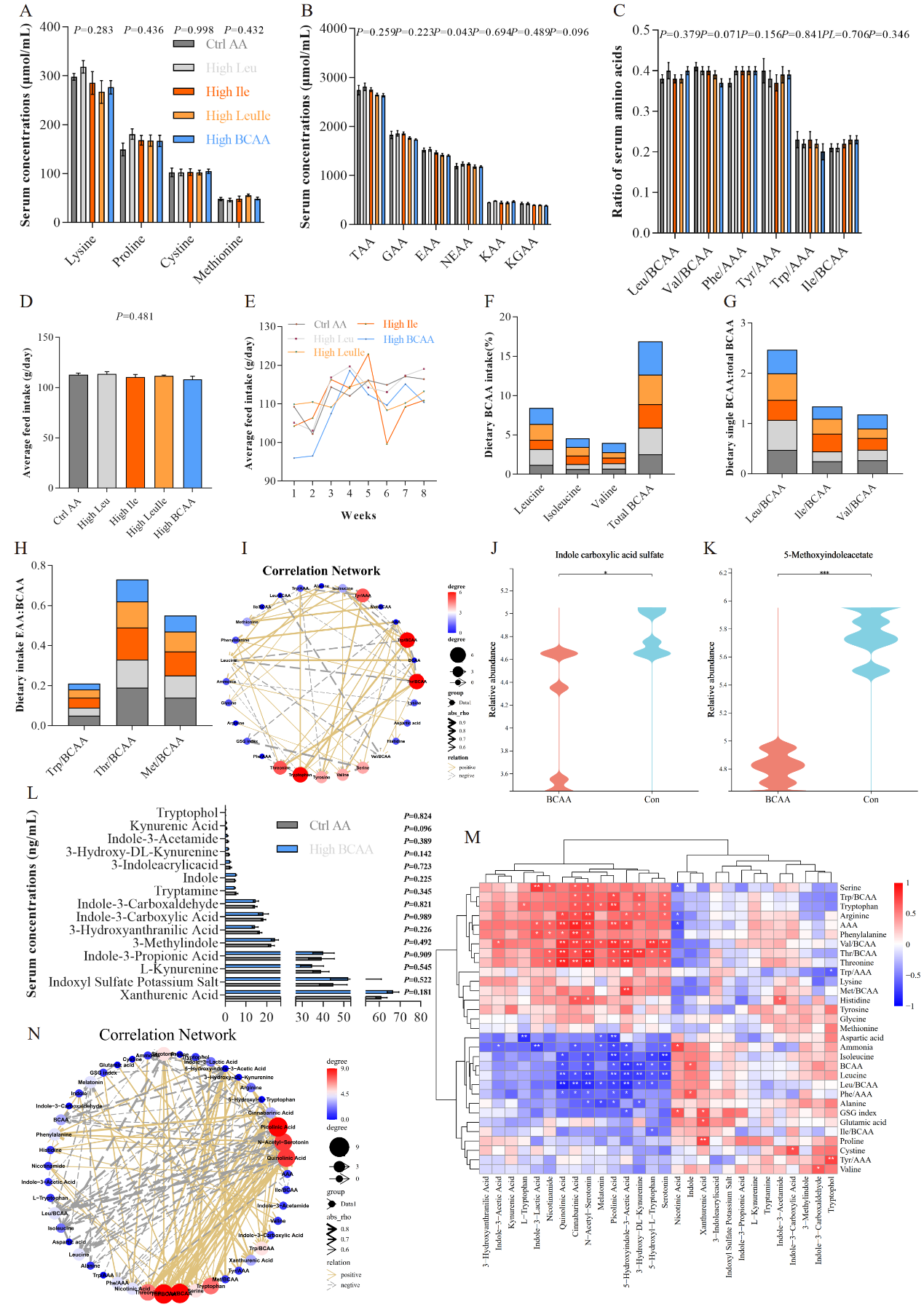


**Fig. S3, related to Fig. 2. High BCAA diet downregulates tryptophan metabolism and leads to indole-3-lactic acid depletion (n=5-6/group).** (A-C) Serum amino acid concentrations and amino acid ratio. (D-E) Feed intakes during High BCAA intervention. (F-H) Average intake of BCAA over 8 weeks (n=36 biologically independent hens). Amino acids were categorized as those that remained stable in intake across diets, BCAA and those that were unstable across diets. (I) Network analysis of serum free amino acids. (J-K) Hepatic tryptophan metabolites abundances from LC-MS/MS in Ctrl AA and High BCAA groups. J, Indole carboxylic acid sulfate. K, 5-Methoxyindoleacetate. (L) The serum metabolites of tryptophan from Ctrl AA and High BCAA groups. (M-N) Heatmap and network Spearman’s correlation analysis of serum free amino acids and tryptophan metabolites from Ctrl AA and High BCAA groups.


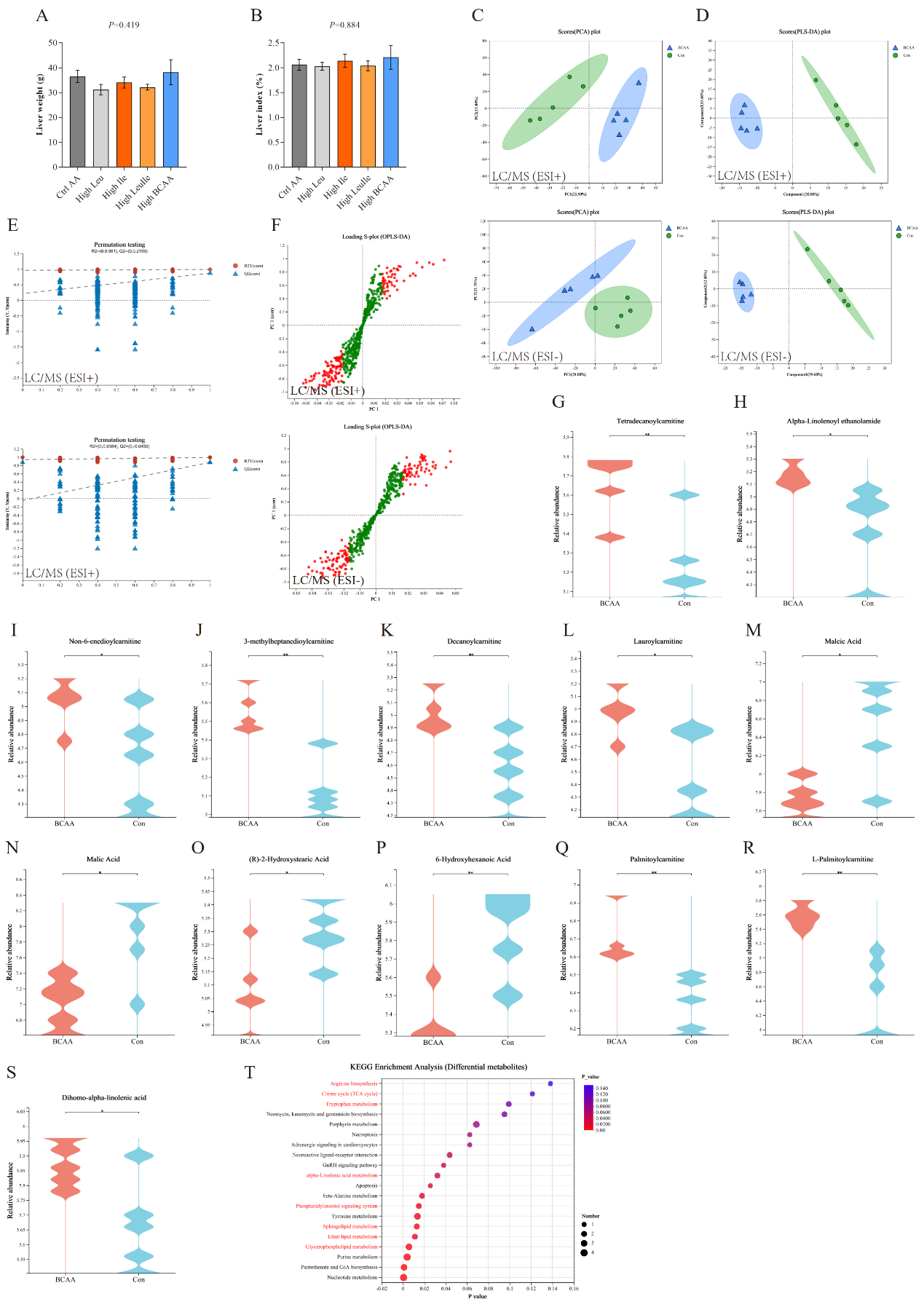


**Fig. S4, related to Fig. 4. High BCAA diet reprograms hepatic lipidome (n=5-6/group).** (A-B) Liver weight and the ratio of liver to body weight. (C-F) PCA, PLS-DA and OPLS-DA analysis of the hepatic metabolome. (G-S) Relative levels of differential metabolites. G, Tetradecanoylcarnitine. H, Alpha-linolenoyl ethanolamide. I, Non-6-enedioylcarnitine. J, 3-Methylheptanedioylcarnitine. K, Decanoylcarnitine. L, Lauroylcarnitine. M, Maleic acid. N, Malic acid. O, (R)-2-Hydroxystearic acid. P, 6-Hydroxyhexanoic Acid. Q, Palmitoylcarnitine. R, L-Palmitoylcarnitine. S, Dihomo-alpha-linolenic acid. (T) KEGG analysis of differential metabolites.


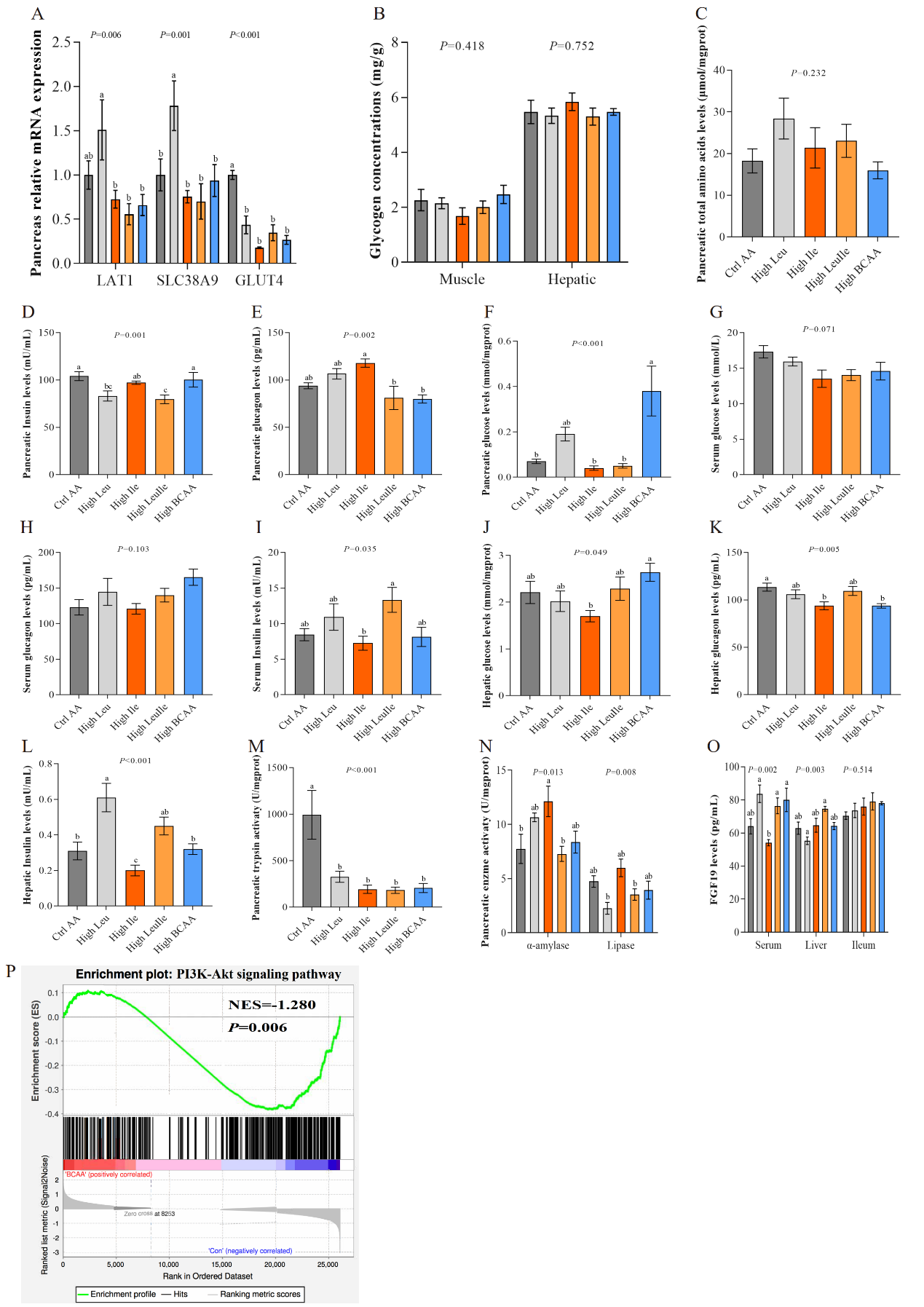


**Fig. S5, related to Fig. 5. Effects of High BCAA diet on glucose and insulin metabolism (n=5-6/group).** (A) Pancreas mRNA expression involved in BCAA and glucose transportation. (B) Hepatic and muscle glycogen contents. (C-F) Pancreatic total amino acids, insulin, glucagon and glucose. (G-I) Serum glucose, glucagon and insulin. (J-L) Hepatic glucose, glucagon and insulin. (M-N) Pancreatic trypsin, α-amylase and lipase activity. (O) FGF19 levels in serum, liver and ileum. (P) GSEA enrichment analysis of PI3K-Akt signaling pathway.


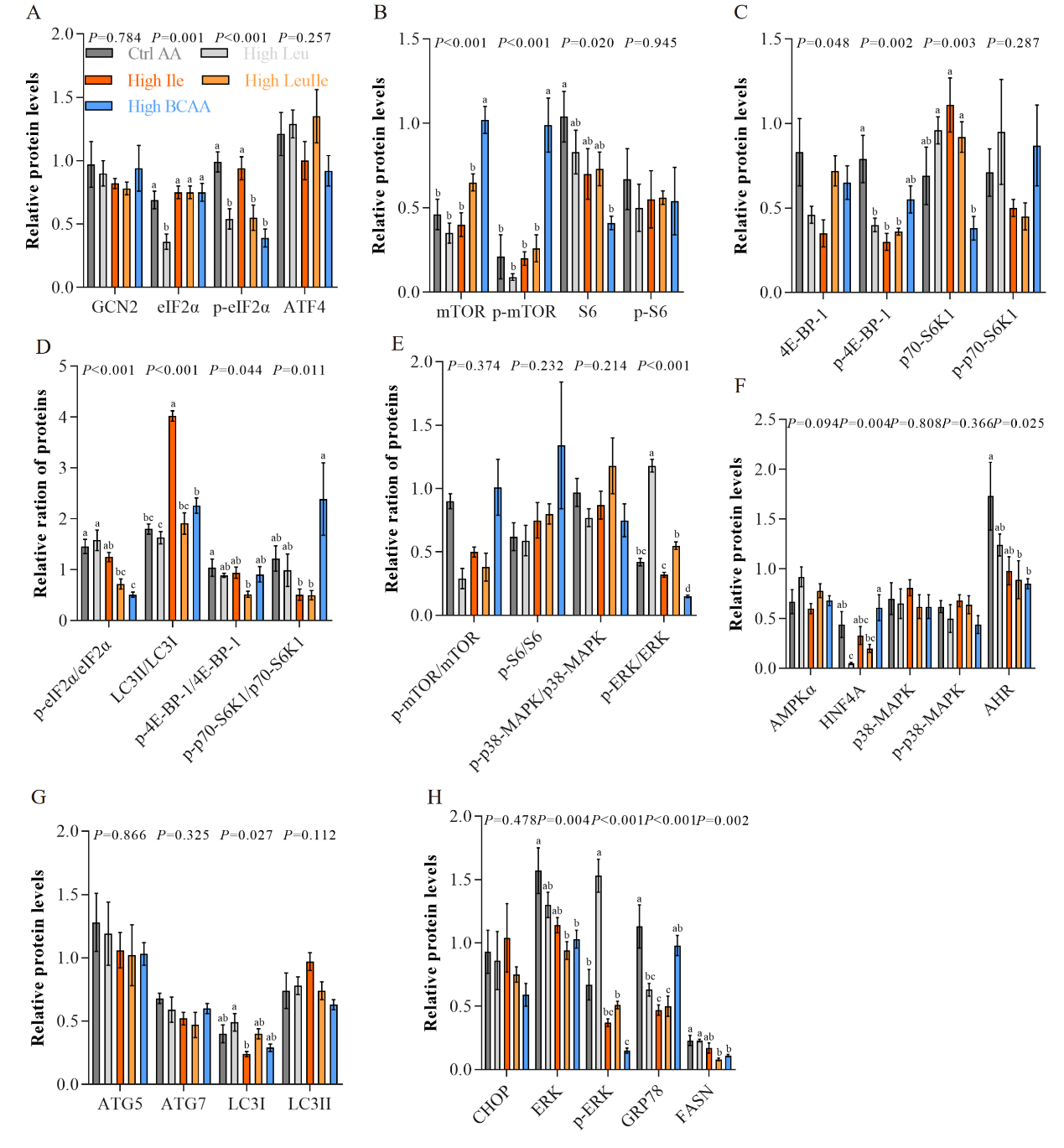


**Fig. S6, related to Fig. 5.** **Hepatic** **GCN2 and mTORC1 are not required for the metabolic effects of a High BCAA diet (n=5/group).** Representative western blot images were quantified once using 5 biologically independent hens. (A) GCN2, eIF2α, phosphorylated eIF2α and ATF4. (B) mTOR, phosphorylated mTOR, S6 and phosphorylated S6. (C) 4E-BP-1, phosphorylated 4E-BP-1, p70-S6K1, phosphorylated p70-S6K1. (D) p-eIF2α/eIF2α, LC3II/ LC3I, p-4E-BP-1/4E-BP-1 and p-p70-S6K1/p70-S6K1. (E) p-mTOR/mTOR, p-S6/S6, p-p38-MAPK/p38-MAPK and p-ERK/ERK. (F) AMPKα, HNF4A, p38-MAPK, p-p38-MAPK and AHR. (G) ATG5, ATG7, LC3I and LC3II. (H) CHOP, ERK, p-ERK, GRP78 and FASN.


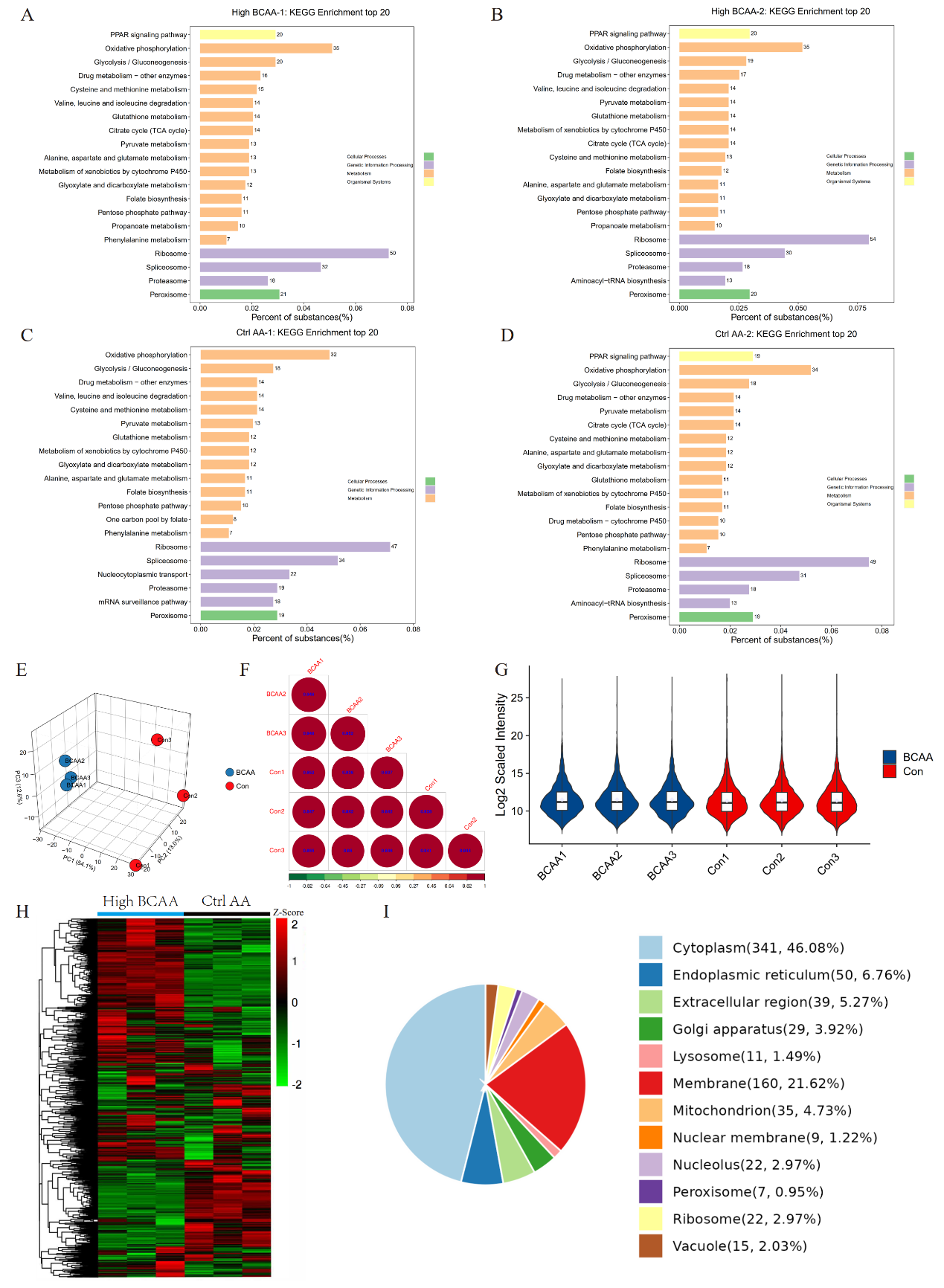


**Fig. S7, related to Fig. 6. High BCAA diet activates MAPK9 ubiquitination by inhibiting UFMylation mediated ubiquitin-proteasome autophagy activation (n=3/group).** (A-D) KEGG functional annotation analysis based on MS analysis using the OECloud tools (n=2/group). (E) Principal component analysis (PCA) of sample from High BCAA and Ctrl AA. (F) Pearson’s correlation coefficients of biological triplicates. (G) Log2 scaled intensity of biological triplicates. (H) Heatmaps of identified ubiquitylation proteins from High BCAA and Ctrl AA. (I) Subcellular localization of identified ubiquitylation proteins from High BCAA and Ctrl AA.


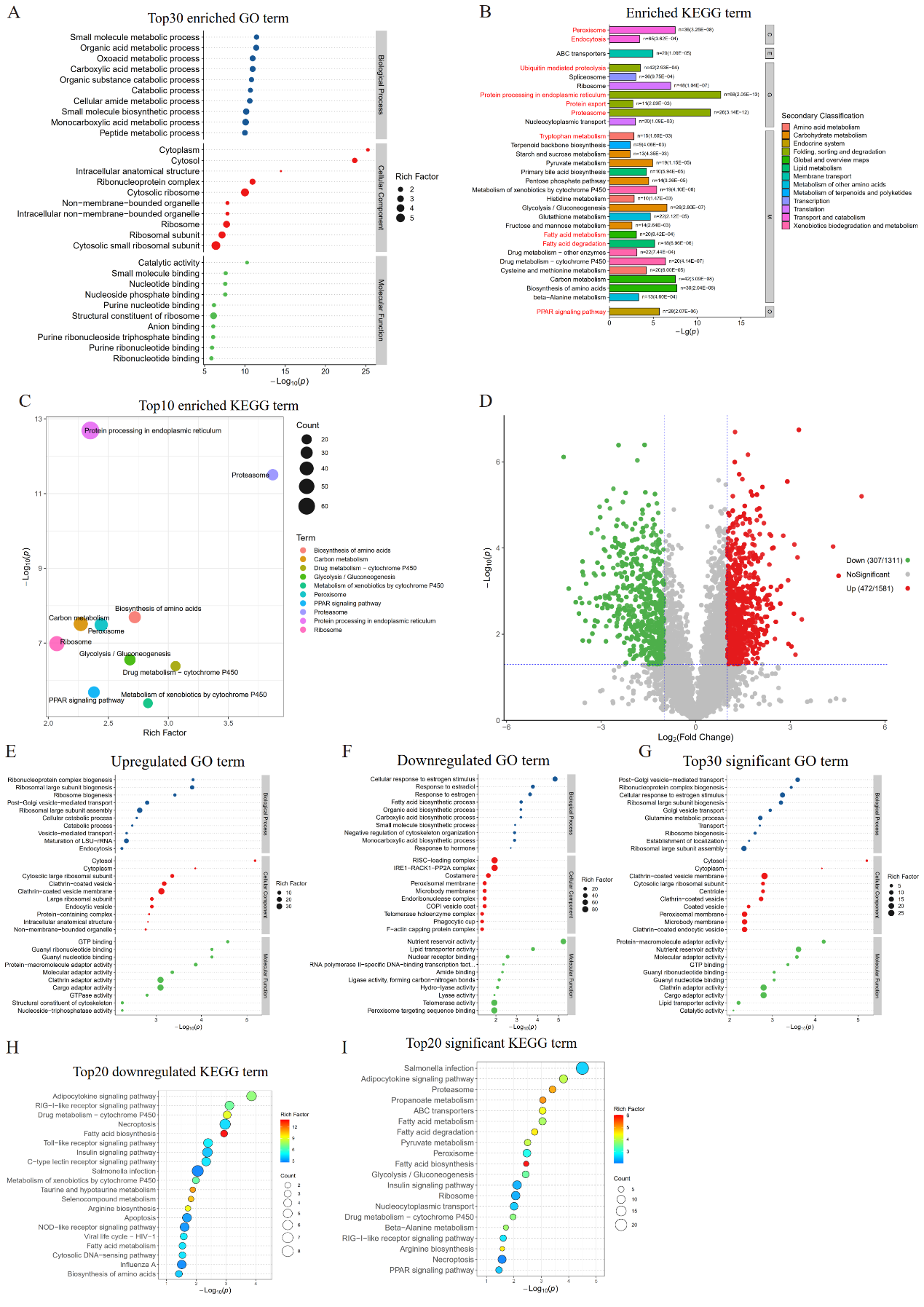


**Fig. S8, related to Fig. 6. Effects of High BCAA diet on ubiquitinating proteome (n=3/group).** (A) GO terms associated with identified ubiquitylation proteins. (B) KEGG terms associated with identified ubiquitylation proteins. (C) Top10 enriched KEGG terms of identified ubiquitylation proteins. (D) Volcano of variable ubiquitination sites in ubiquitin-modified proteome analysis of Ctrl AA or High BCAA-fed hens livers. (E) GO terms associated with identified upregulated ubiquitination sites proteins. (F) GO terms associated with identified downregulated ubiquitination sites proteins. (G) GO terms associated with identified significant ubiquitination sites proteins. (H) Top20 KEGG terms associated with identified downregulated ubiquitination sites proteins. (I) Top20 KEGG terms associated with identified variable ubiquitination sites proteins.


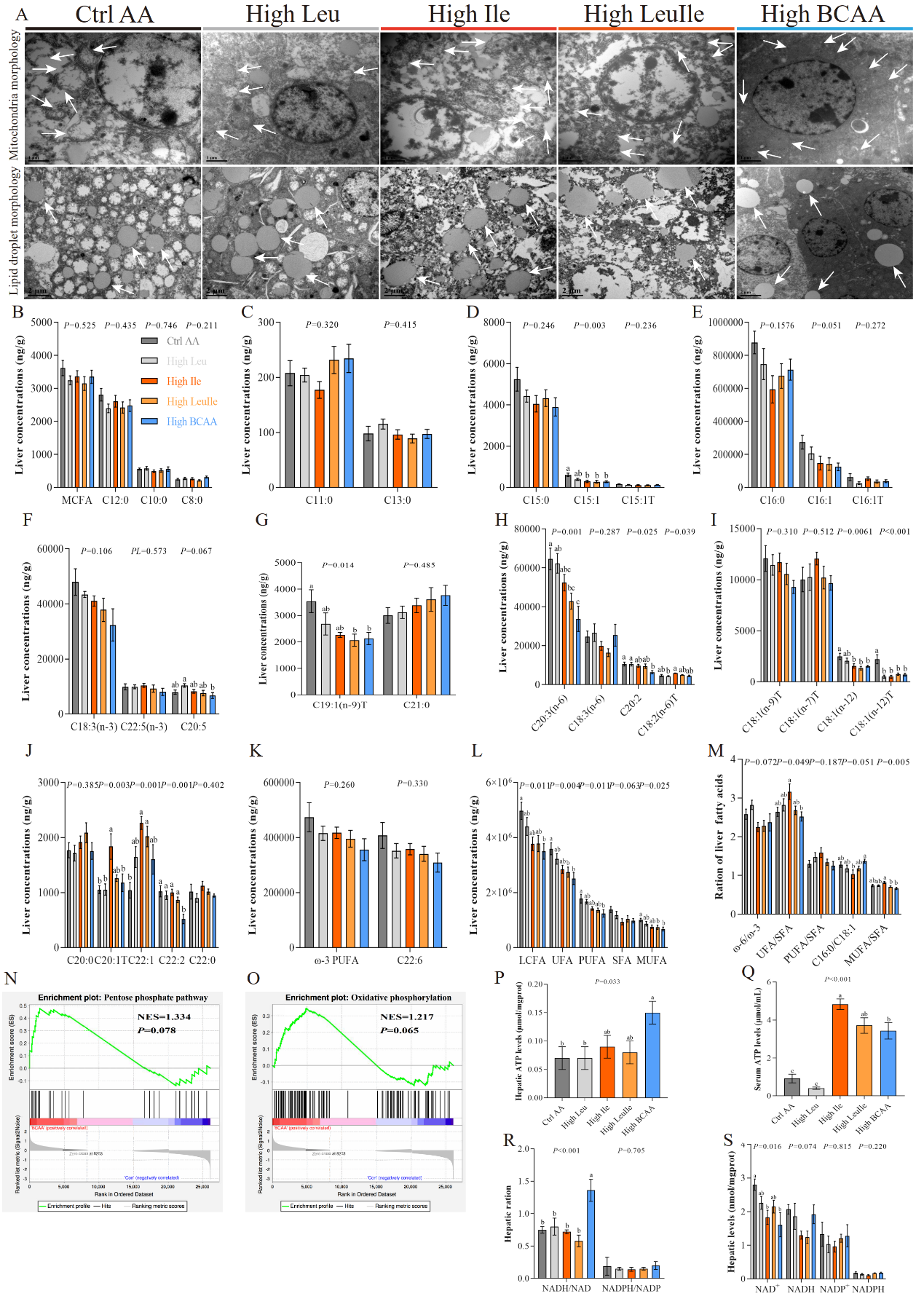


**Fig. S9, related to Fig. 7. High BCAA diet promotes hepatic lipid catabolism by activating PPAR-RXR (n=5-6/group).** (A) Hepatic mitochondrial and lipid droplet ultrastructural of TEM (n=3). (B-M) Hepatic fatty acid levels. (N) GSEA enrichment analysis of pentose phosphate pathway. (O) GSEA enrichment analysis of oxidative phosphorylation pathway. (P) Hepatic ATP levels. (Q) Serum ATP levels. (R) Hepatic NAD, NADH, NADP and NADPH levels. (S) Hepatic ratio of NAD/NADH and NADP/NADPH.


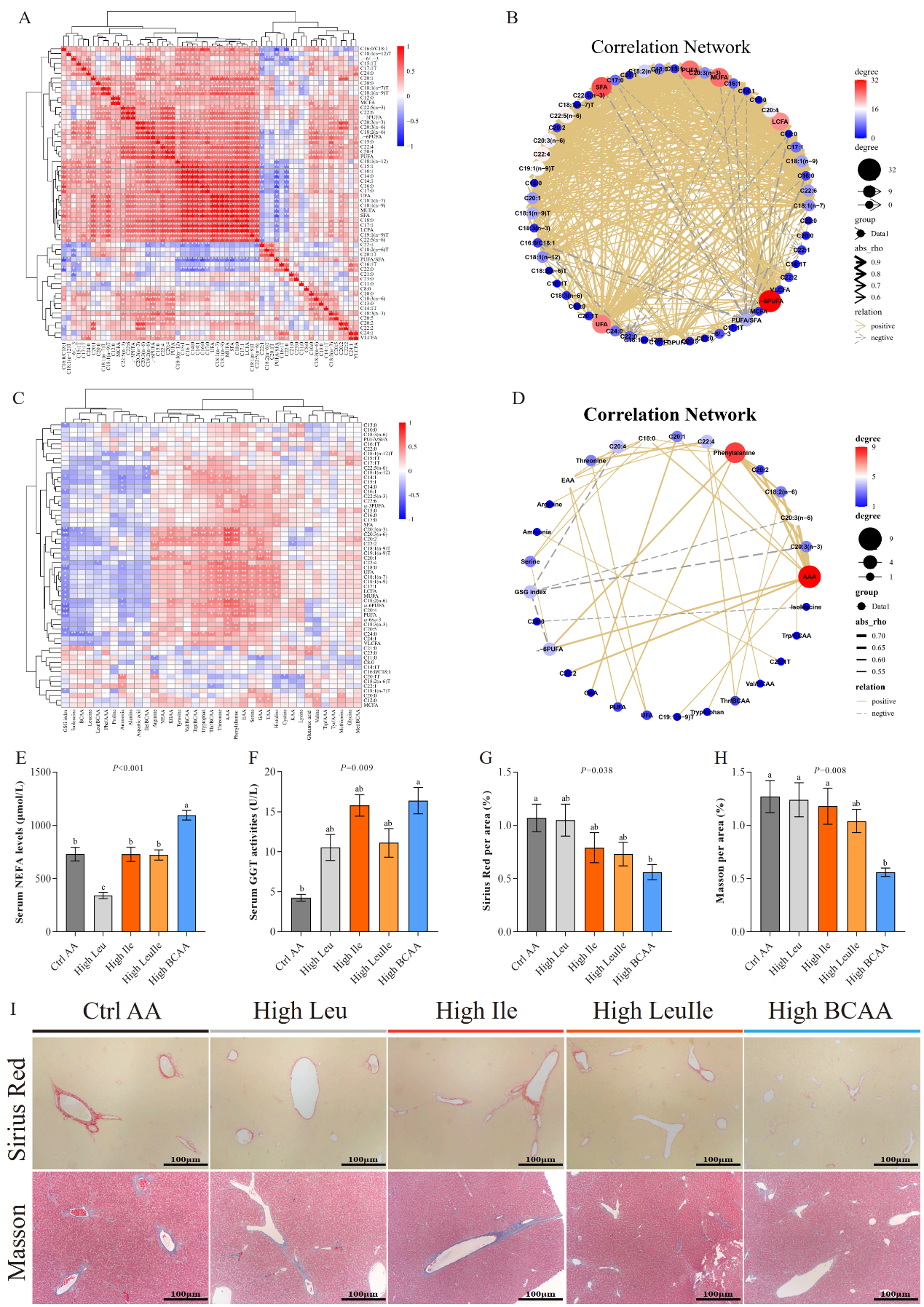


**Fig. S10, related to Fig. 8. Effects of High BCAA diet on hepatic fatty acid and amino acid (n=5-6/group).** (A-B) Hepatic fatty acid Spearman’s correlation analysis. (C-D) Serum amino acid and hepatic fatty acid Spearman’s correlation analysis. (E) Serum NEFA. (F) Serum GGT. (G-H) Hepatic Masson staining and Sirius staining area. (I) Representative images of hepatic Masson staining and Sirius staining.


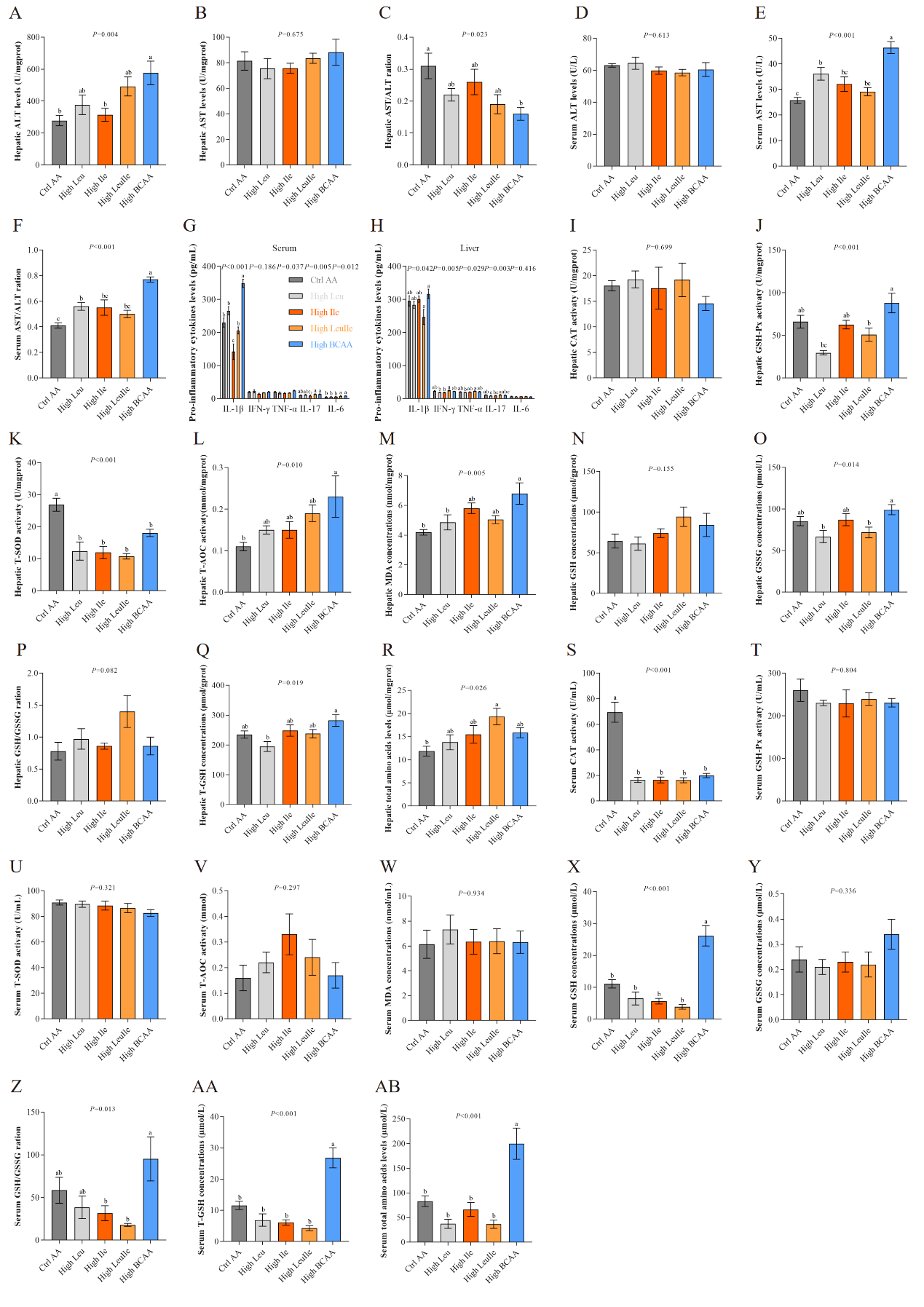


**Fig. S11, related to Fig. 8. Effects of High BCAA diet on inflammatory response and oxidative stress (n=5-6/group).** (A-C) Hepatic ALT, AST and AST/ALT. (D-G) Serum ALT, AST, AST/ALT and pro-inflammatory cytokines. (H-R) Hepatic pro-inflammatory cytokines, CAT, GSH-Px, T-SOD, T-AOC, MDA, GSH, GSSG, GSH/GSSG, T-GSH and total amino acids. (S-AB) Serum CAT, GSH-Px, T-SOD, T-AOC, MDA, GSH, GSSG, GSH/GSSG, T-GSH and total amino acids.


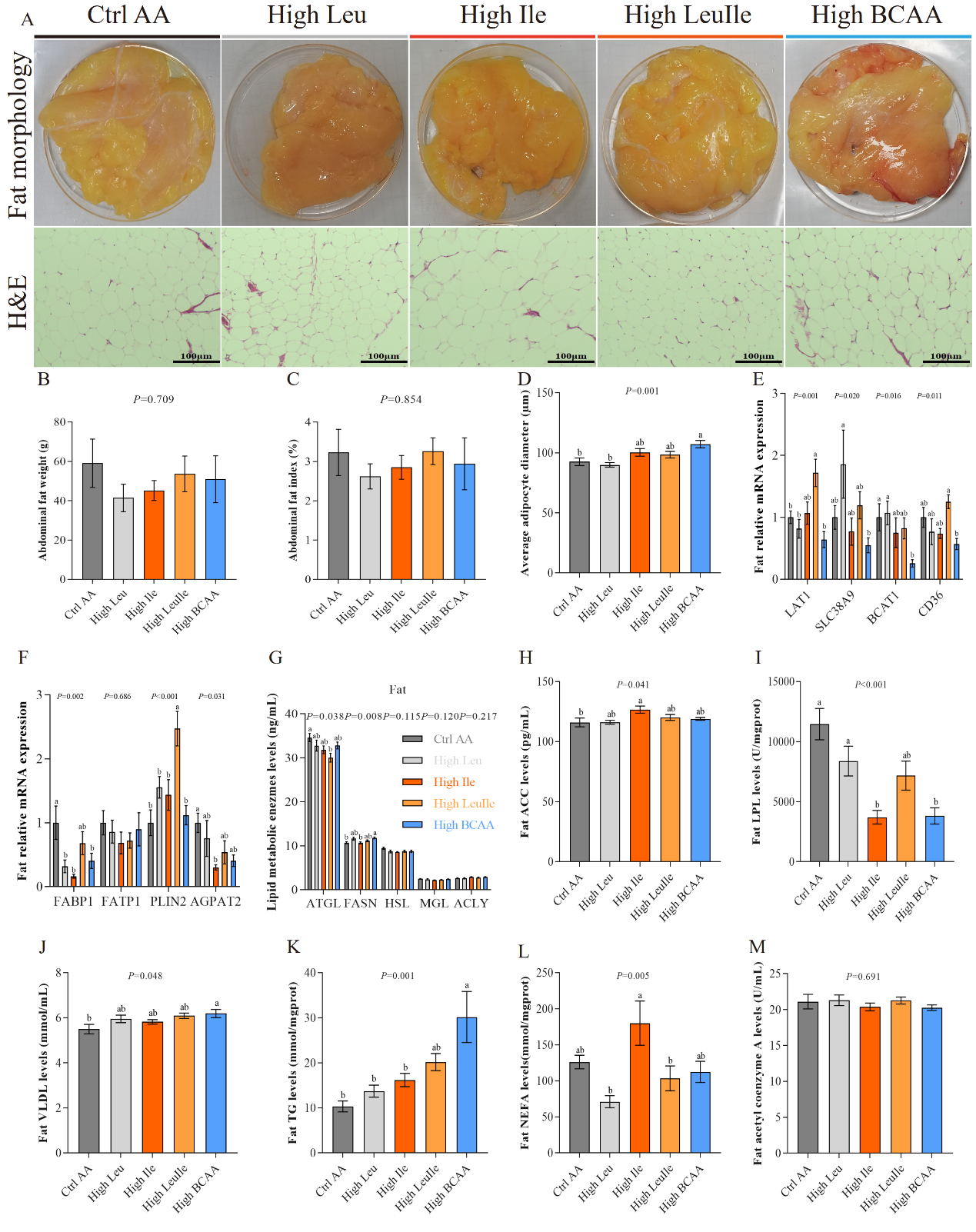


**Fig. S12. Effects of High BCAA diet on lipid metabolism in adipose tissues.** (A) Abdominal fat H&E straining. (B) Abdominal fat weight. (C) Abdominal fat index. (D) Average adipocyte diameter. (E-F) Abdominal fat relative mRNA expression. (G-I) Abdominal fat lipid metabolism related enzyme levels. (J) Abdominal fat VLDL level. (K) Abdominal fat TG level. (L) Abdominal fat NEFA level. (M) Abdominal fat acetyl-CoA level.
